# Supplementary material for: TIVelo: RNA velocity estimation leveraging cluster-level trajectory inference
Source: Nat Commun. 2025 Jul 7;16:6258. doi: 10.1038/s41467-025-61628-x (PMC12234748; doi:10.1038/s41467-025-61628-x)
Supplement: Supplementary file 1 — Supplementary Information [file 41467_2025_61628_MOESM1_ESM.pdf]

Supplementary Information of "TIVelo: RNA velocity estimation leveraging cluster-level trajectory inference"

Muyang Ge[1], Jishuai Miao[1], Ji Qi[1], Xiaocheng Zhou[1], and Zhixiang Lin[1,*]

[1]Department of Statistics, The Chinese University of Hong Kong, Shatin, Hong Kong SAR, China
[*]Correspondence: zhixianglin@cuhk.edu.hk

Supplementary Figures

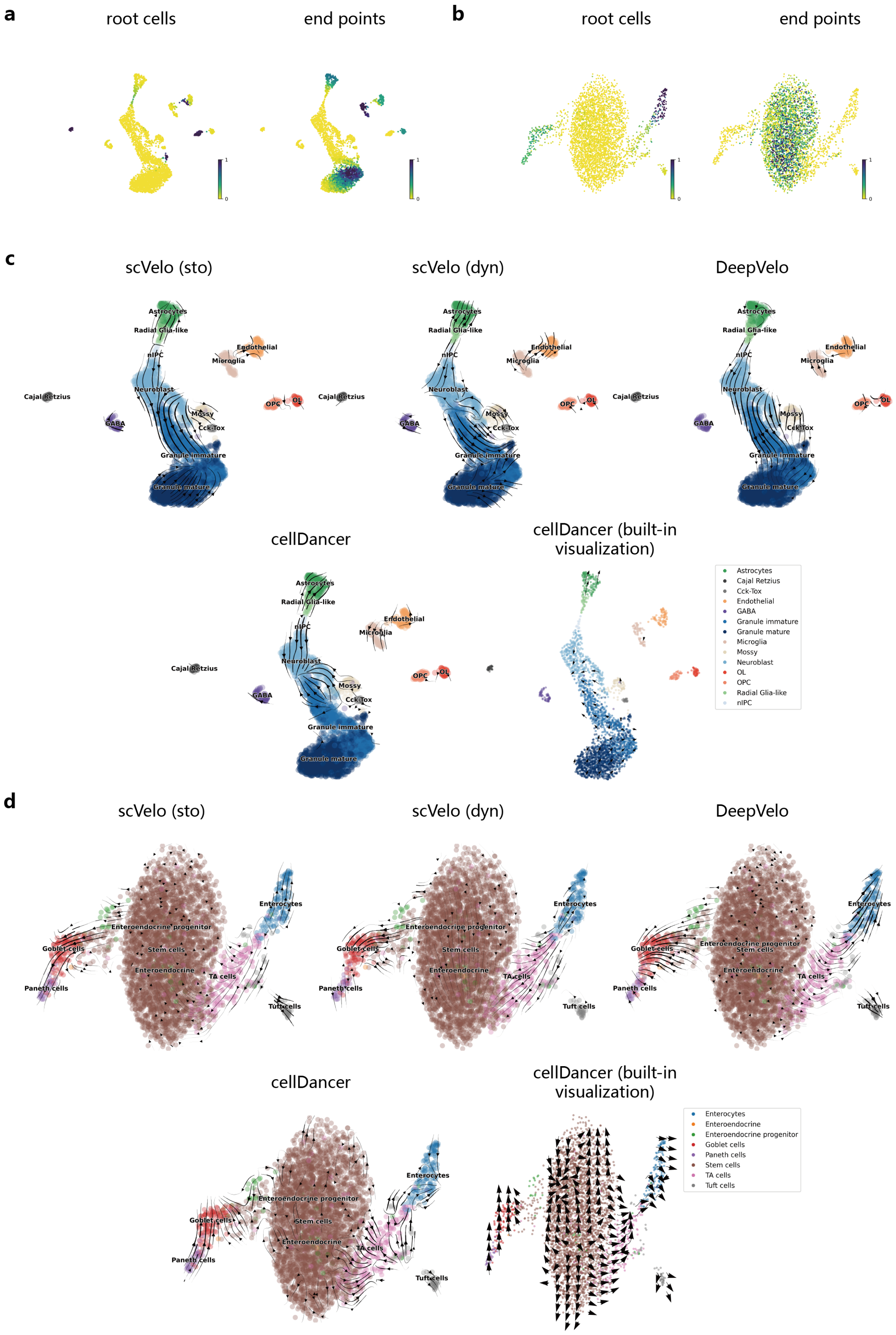

Supplementary Fig. 1 | Supplementary results from dentate gyrus development and intestinal organoid. **a**. The root cells and end points scores inferred from scVelo on dentate gyrus development. **b**. The root cells and end points scores inferred from scVelo on intestinal organoid. **c**. Comparative velocity stream plots of dentate gyrus development produced by scVelo (stochastic mode), scVelo (dynamical mode), DeepVelo, cellDancer (visualization by scVelo) and cellDancer (visualization by built-in method in cellDancer). **c**. Comparative velocity stream plots of intestinal organoid produced by scVelo (stochastic mode), scVelo (dynamical mode), DeepVelo, cellDancer (visualization by scVelo) and cellDancer (visualization by built-in method in cellDancer).

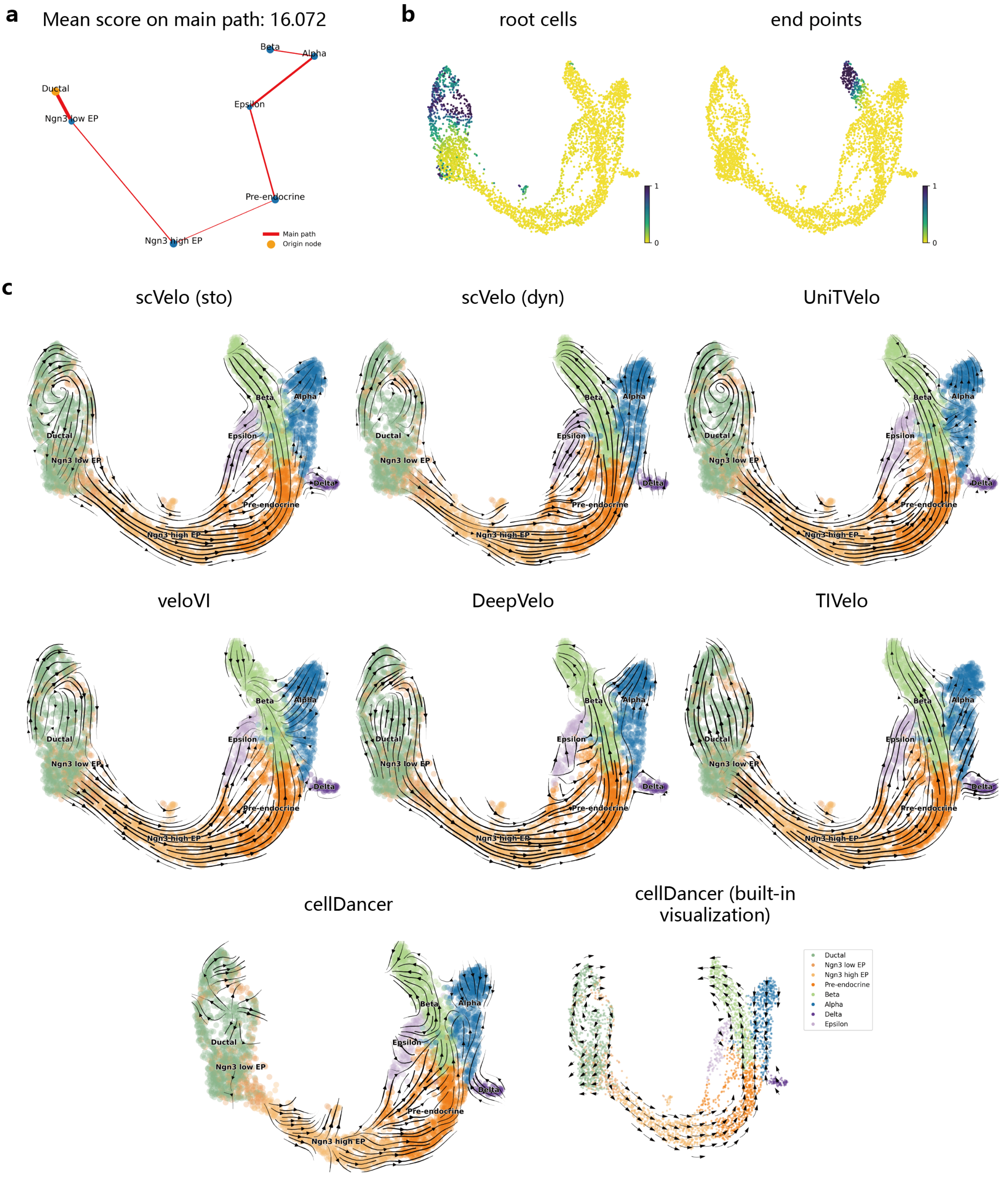

Supplementary Fig. 2 | Evaluation of TIVelo's performance on pancreatic endocrinogenesis. **a**. The cluster graph after graph pruning and main path selection. The cluster Ductal is selected as the origin node. The mean orientation score along the main path is 16.072. **b**. The root cells and end points scores inferred from scVelo. **c**. Comparative velocity stream plots of pancreatic endocrinogenesis produced by scVelo (stochastic mode), scVelo (dynamical mode), UniTVelo, veloVI, DeepVelo, TIVelo, cellDancer (visualization by scVelo) and cellDancer (visualization by built-in method in cellDancer).

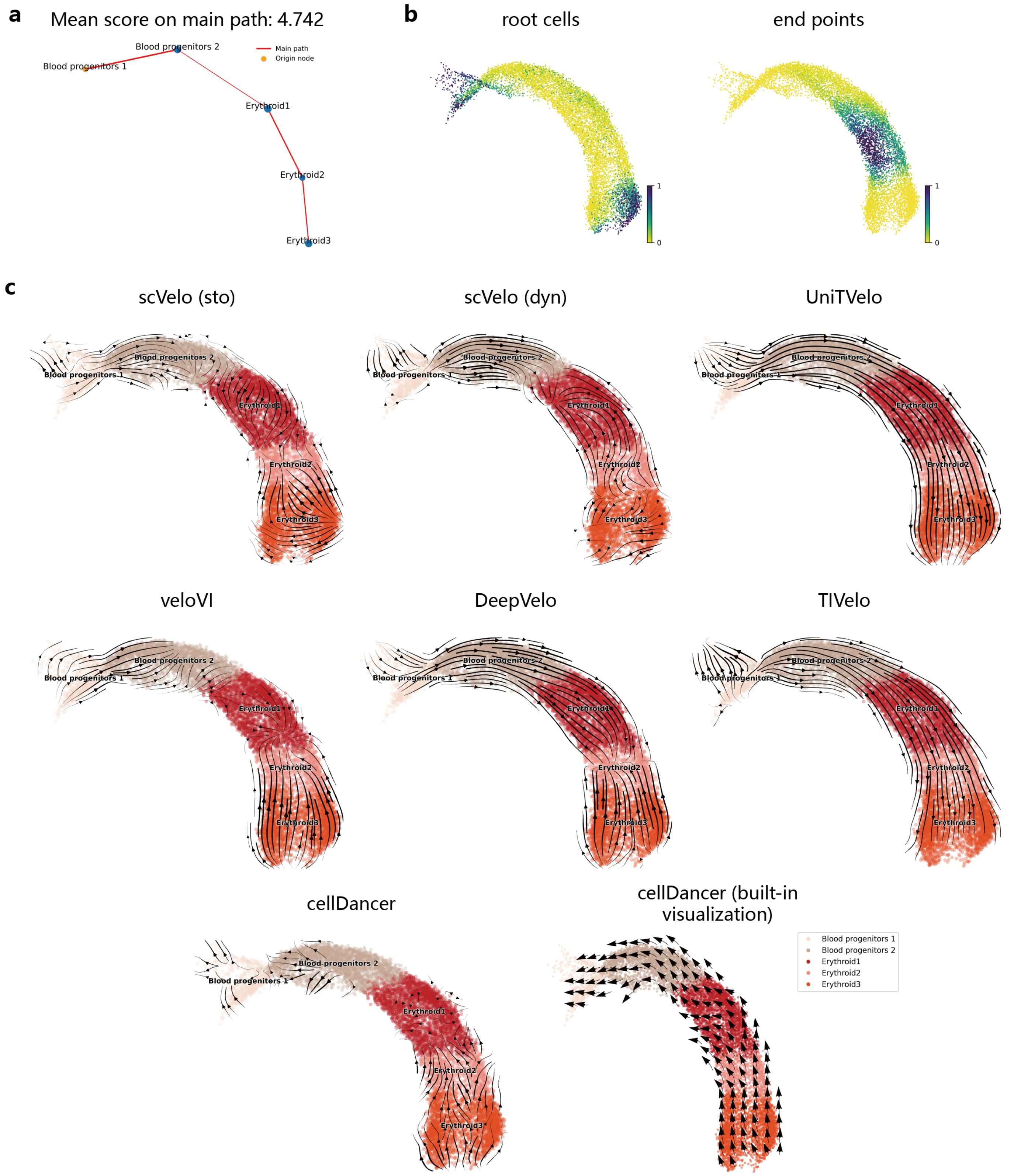

Supplementary Fig. 3 | Evaluation of TIVelo's performance on mouse gastrulation (erythroid). **a**. The cluster graph after graph pruning and main path selection. The cluster Blood Progenitors 1 is selected as the origin node. The mean orientation score along the main path is 4.742. **b**. The root cells and end points scores inferred from scVelo. **c**. Comparative velocity stream plots of mouse gastrulation produced by scVelo (stochastic mode), scVelo (dynamical mode), UniTVelo, veloVI, DeepVelo, TIVelo, cellDancer (visualization by scVelo) and cellDancer (visualization by built-in method in cellDancer).

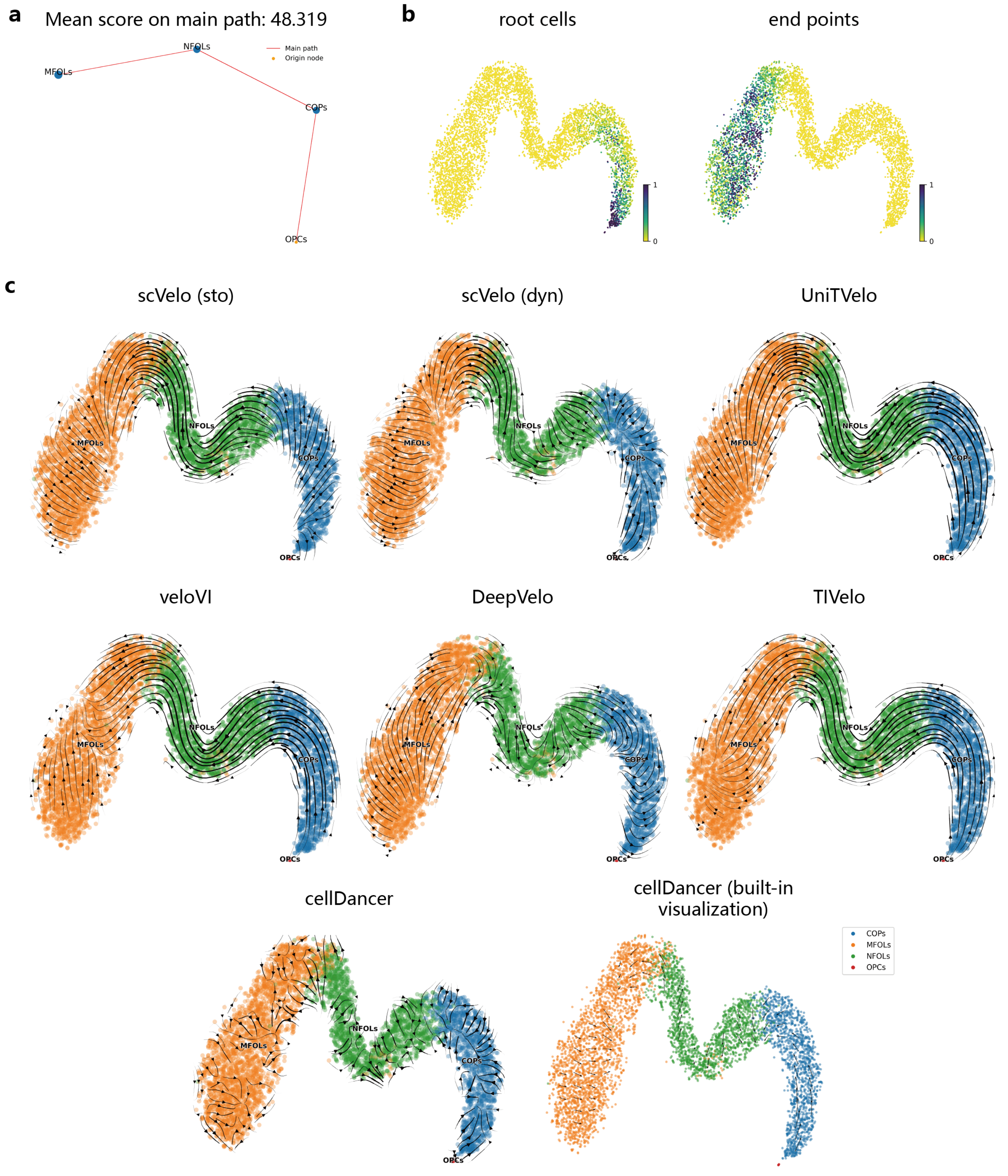

Supplementary Fig. 4 | Evaluation of TIVelo's performance on mouse hindbrain (Oligo). **a**. The cluster graph after graph pruning and main path selection. The cluster OPCs is selected as the origin node. The mean orientation score along the main path is 48.319. **b**. The root cells and end points scores inferred from scVelo. **c**. Comparative velocity stream plots of mouse hindbrain (Oligo) produced by scVelo (stochastic mode), scVelo (dynamical mode), UniTVelo, veloVI, DeepVelo, TIVelo, cellDancer (visualization by scVelo) and cellDancer (visualization by built-in method in cellDancer).

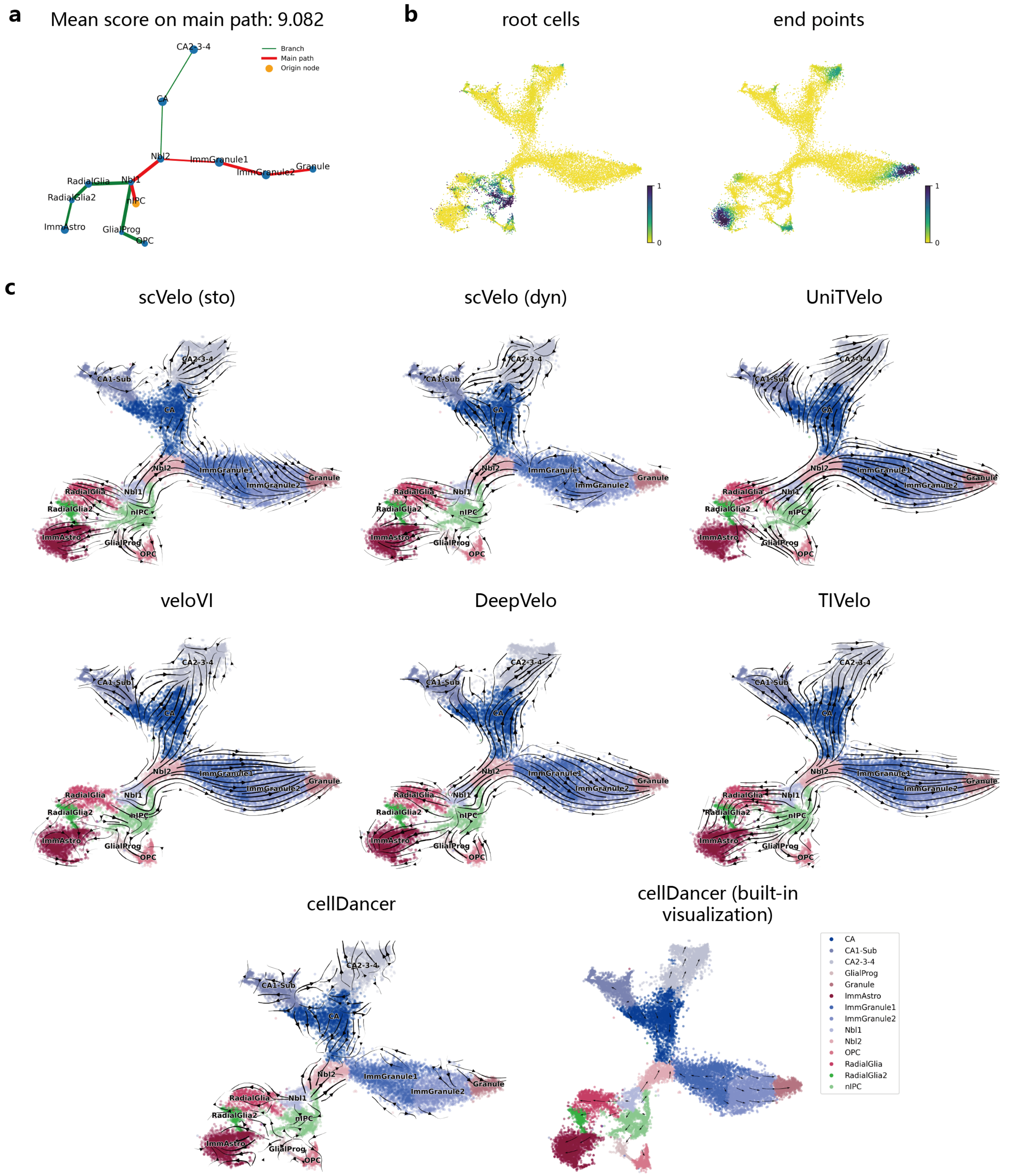

Supplementary Fig. 5 | Evaluation of TIVelo's performance on dentate gyrus development 2. a. The cluster graph after graph pruning and main path selection. The cluster nIPC is selected as the origin node. The mean orientation score along the main path is 9.082. **b**. The root cells and end points scores inferred from scVelo. **c**. Comparative velocity stream plots of dentate gyrus development 2 produced by scVelo (stochastic mode), scVelo (dynamical mode), UniTVelo, veloVI, DeepVelo, TIVelo, cellDancer (visualization by scVelo) and cellDancer (visualization by built-in method in cellDancer).

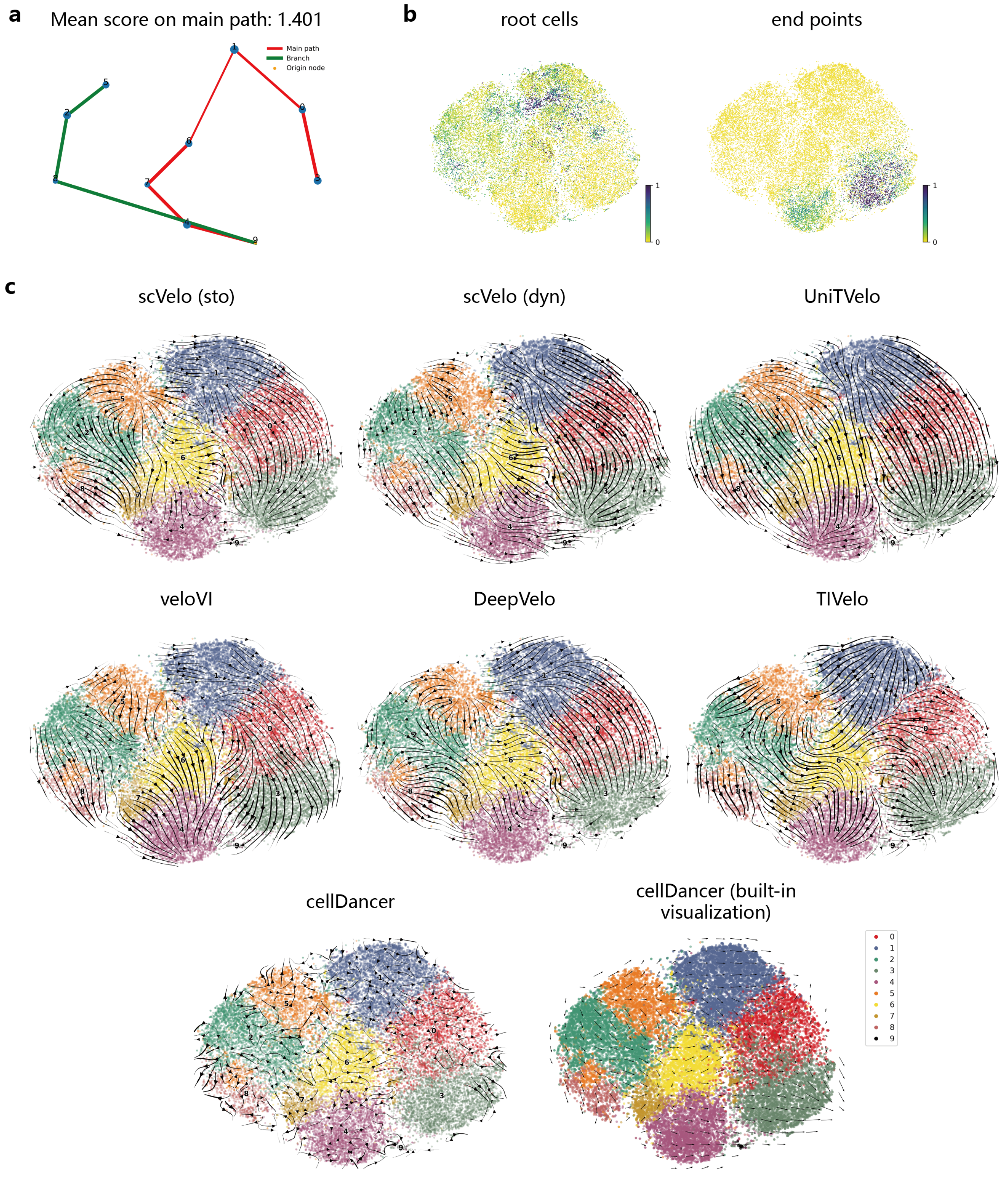

Supplementary Fig. 6 | Evaluation of TIVelo's performance on mouse embryonic fibroblast reprogramming. **a**. The cluster graph after graph pruning and main path selection. The cluster 9 is selected as the origin node. The mean orientation score along the main path is 1.401. The mean orientation score along the branch in the graph is -0.972. Since this branch connects to the main path both by their endpoints, the origin node is reset as cluster 5 (Methods; Supplementary Fig. 14). **b**. The root cells and end points scores inferred from scVelo. **c**. Comparative velocity stream plots of mouse embryonic fibroblast reprogramming produced by scVelo (stochastic mode), scVelo (dynamical mode), UniTVelo, veloVI, DeepVelo, TIVelo, cellDancer (visualization by scVelo) and cellDancer (visualization by built-in method in cellDancer).

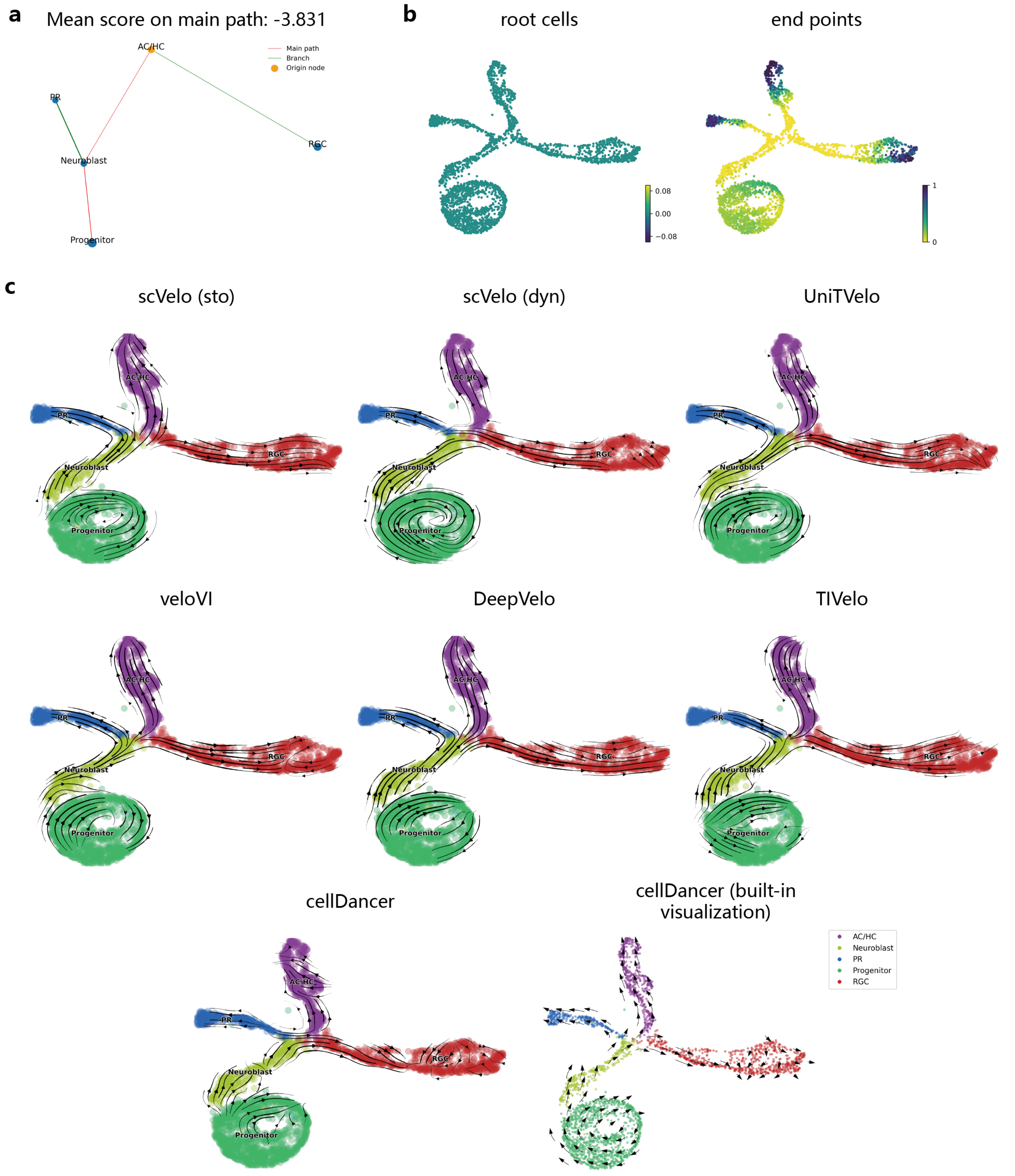

Supplementary Fig. 7 | Evaluation of TIVelo's performance on mouse retina development. **a**. The cluster graph after graph pruning and main path selection. The cluster AC/HC is selected as the origin node. The mean orientation score along the main path is -3.831. The origin node is reset as cluster Progenitor. **b**. The root cells and end points scores inferred from scVelo. **c**. Comparative velocity stream plots of mouse retina development produced by scVelo (stochastic mode), scVelo (dynamical mode), UniTVelo, veloVI, DeepVelo, TIVelo, cellDancer (visualization by scVelo) and cellDancer (visualization by built-in method in cellDancer).

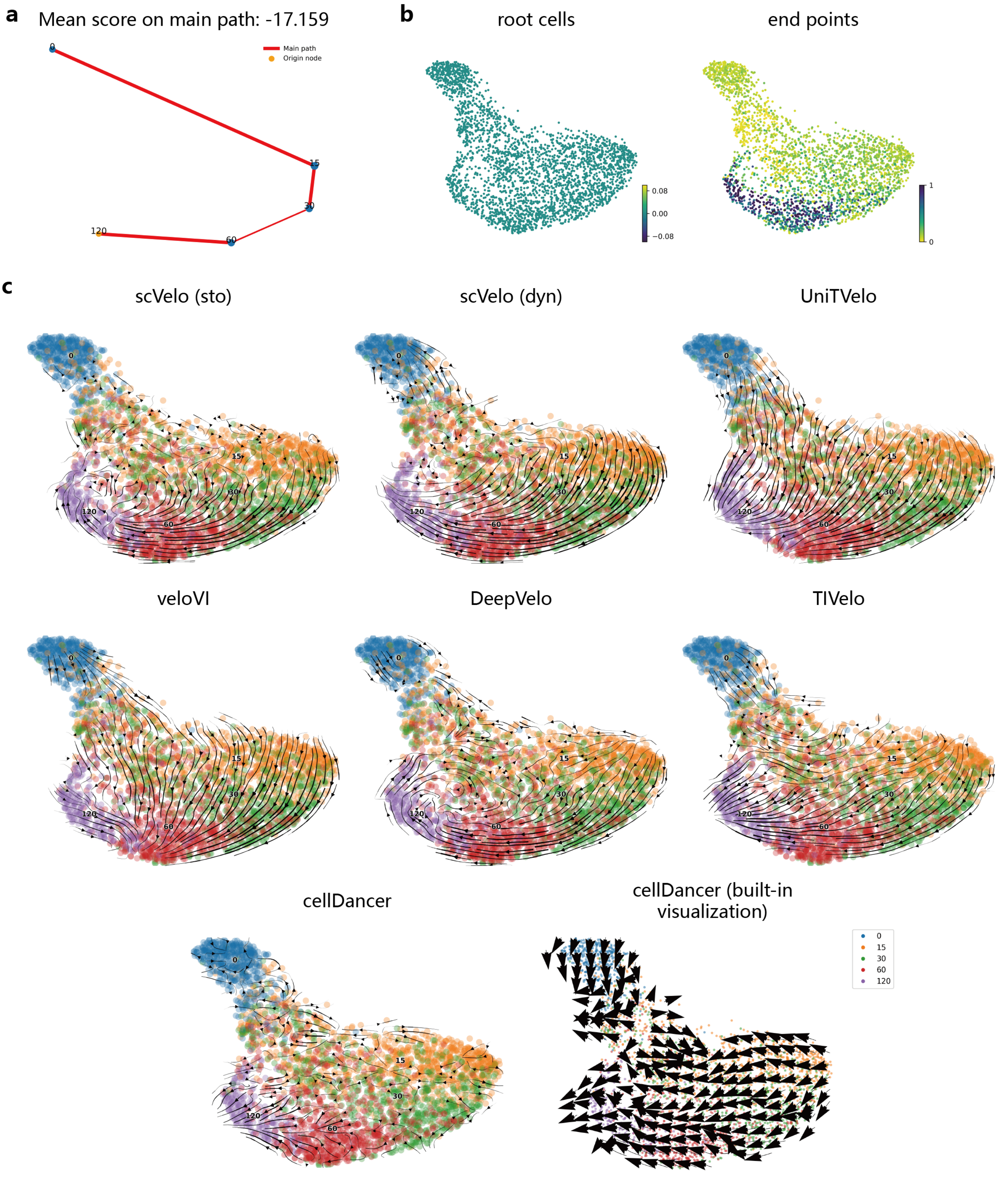

Supplementary Fig. 8 | Evaluation of TIVelo's performance on scNT-seq neuron KCl stimulation. **a**. The cluster graph after graph pruning and main path selection. The cluster 120 is selected as the origin node. The mean orientation score along the main path is -17.159. The origin node is reset as cluster 0. **b**. The root cells and end points scores inferred from scVelo. **c**. Comparative velocity stream plots of scNT-seq neuron KCl stimulation produced by scVelo (stochastic mode), scVelo (dynamical mode), UniTVelo, veloVI, DeepVelo, TIVelo, cellDancer (visualization by scVelo) and cellDancer (visualization by built-in method in cellDancer).

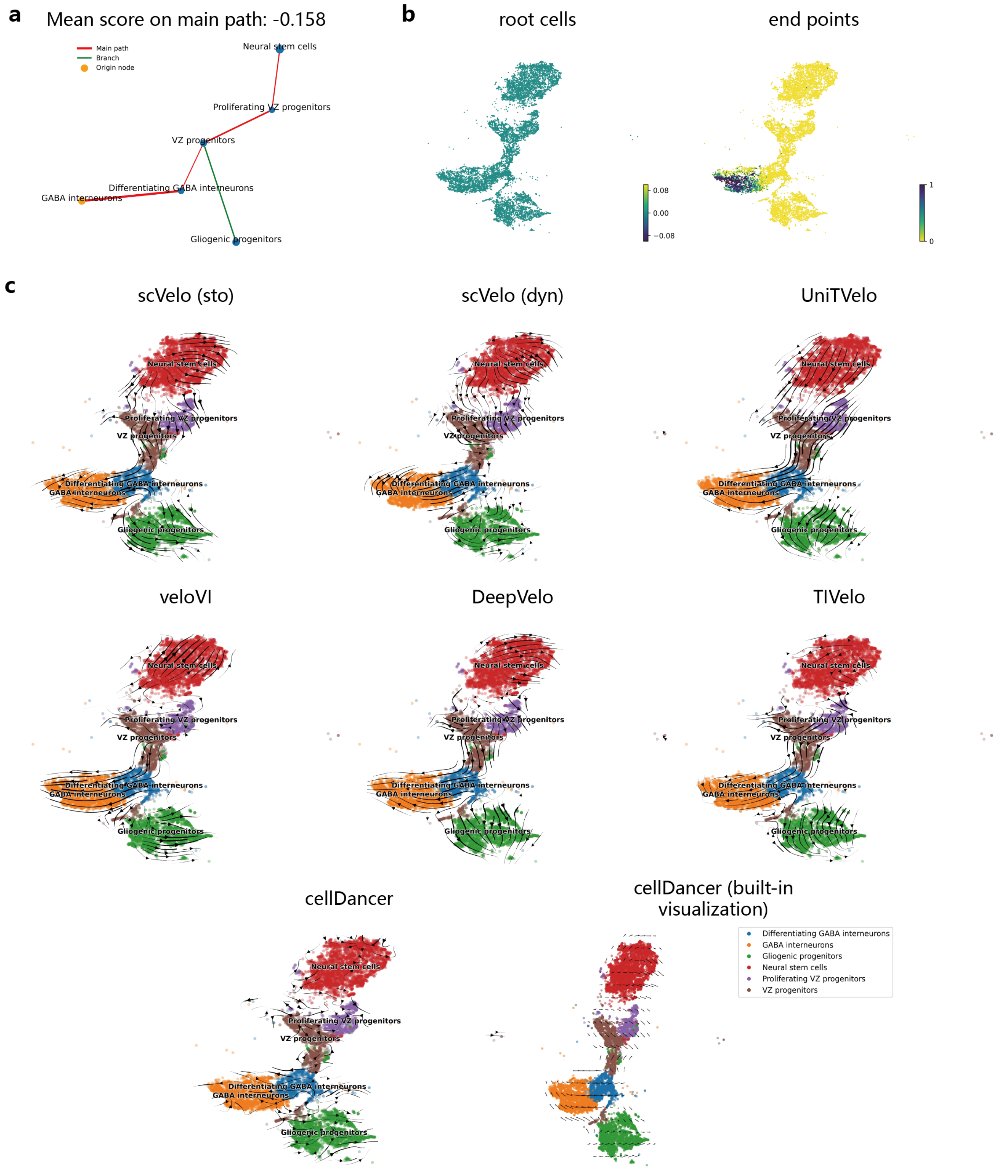

Supplementary Fig. 9 | Evaluation of TIVelo's performance on mouse hindbrain (GABA, Glial). **a**. The cluster graph after graph pruning and main path selection. The cluster GABA interneurons is selected as the origin node. The mean orientation score along the main path is -0.158. The origin node is reset as cluster Neural stem cells. **b**. The root cells and end points scores inferred from scVelo. **c**. Comparative velocity stream plots of mouse hindbrain (GABA, Glial) produced by scVelo (stochastic mode), scVelo (dynamical mode), UniTVelo, veloVI, DeepVelo, TIVelo, cellDancer (visualization by scVelo) and cellDancer (visualization by built-in method in cellDancer).

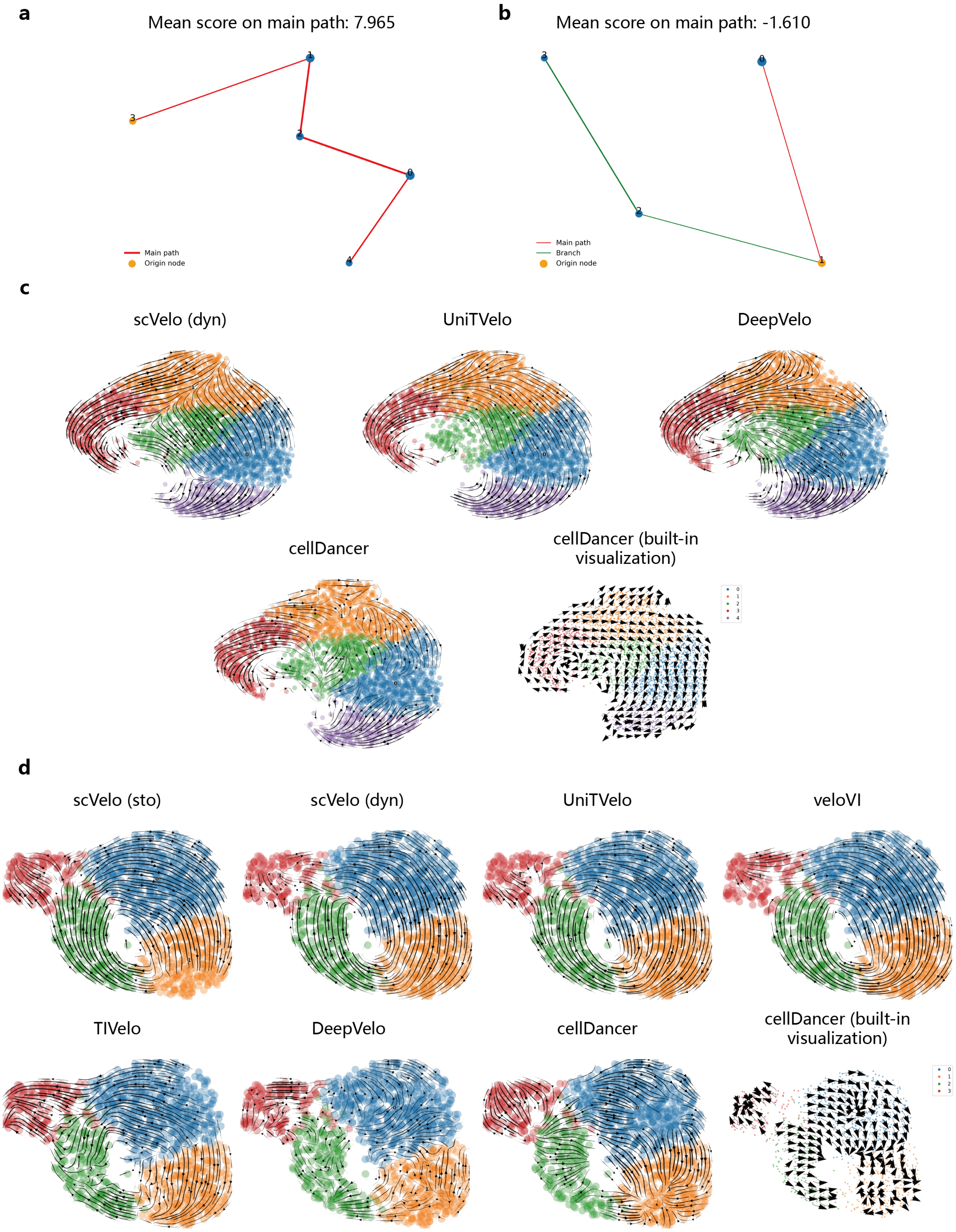
a
Mean score on main path: 7.965
Main path
Origin node
b
Mean score on main path: -1.610
Main path
Branch
Origin node
c
scVelo (dyn)
UniTVelo
DeepVelo
cellDancer
cellDancer (built-in visualization)
d
scVelo (sto)
scVelo (dyn)
UniTVelo
veloVI
TIVelo
DeepVelo
cellDancer
cellDancer (built-in visualization)

Supplementary Fig. 10 | Supplementary results on two datasets with cell-cycle scores. **a**. The cluster graph after graph pruning and main path selection on RPE1-FUCCI. The cluster 3 is selected as the origin node. The mean orientation score along the main path is 7.965. **b**. The cluster graph after graph pruning and main path selection on U2OS-FUCCI. The cluster 1 is selected as the origin node. The mean orientation score along the main path is -1.610. The origin node is reset as cluster 0. **c**. Comparative velocity stream plots of RPE1-FUCCI produced by scVelo (dynamical mode), UniTVelo, DeepVelo, cellDancer (visualization by scVelo) and cellDancer (visualization by built-in method in cellDancer). **d**. Comparative velocity stream plots of U2OS-FUCCI produced by scVelo (stochastic mode), scVelo (dynamical mode), UniTVelo, veloVI, TIVelo, DeepVelo, cellDancer (visualization by scVelo) and cellDancer (visualization by built-in method in cellDancer). For U2OS-FUCCI, the velocity stream is expected to show an anticlockwise cycling pattern, as in the result from veloVI. While TIVelo successfully captures the overall velocity direction at the cluster level (cluster $0 \rightarrow 1 \rightarrow 2 \rightarrow 3$), its current implementation is limited to tree-structured cluster graphs. This structural constraint leads to suboptimal prediction of the developmental transition from cluster 3 back to cluster 0, resulting in some reversed velocity stream from cluster 0 to cluster 2 and 3. Nevertheless, TIVelo achieves highest velocity sign accuracy (Fig. 5e), primarily because the trajectory cluster $0 \rightarrow 1 \rightarrow 2$ is correctly inferred.

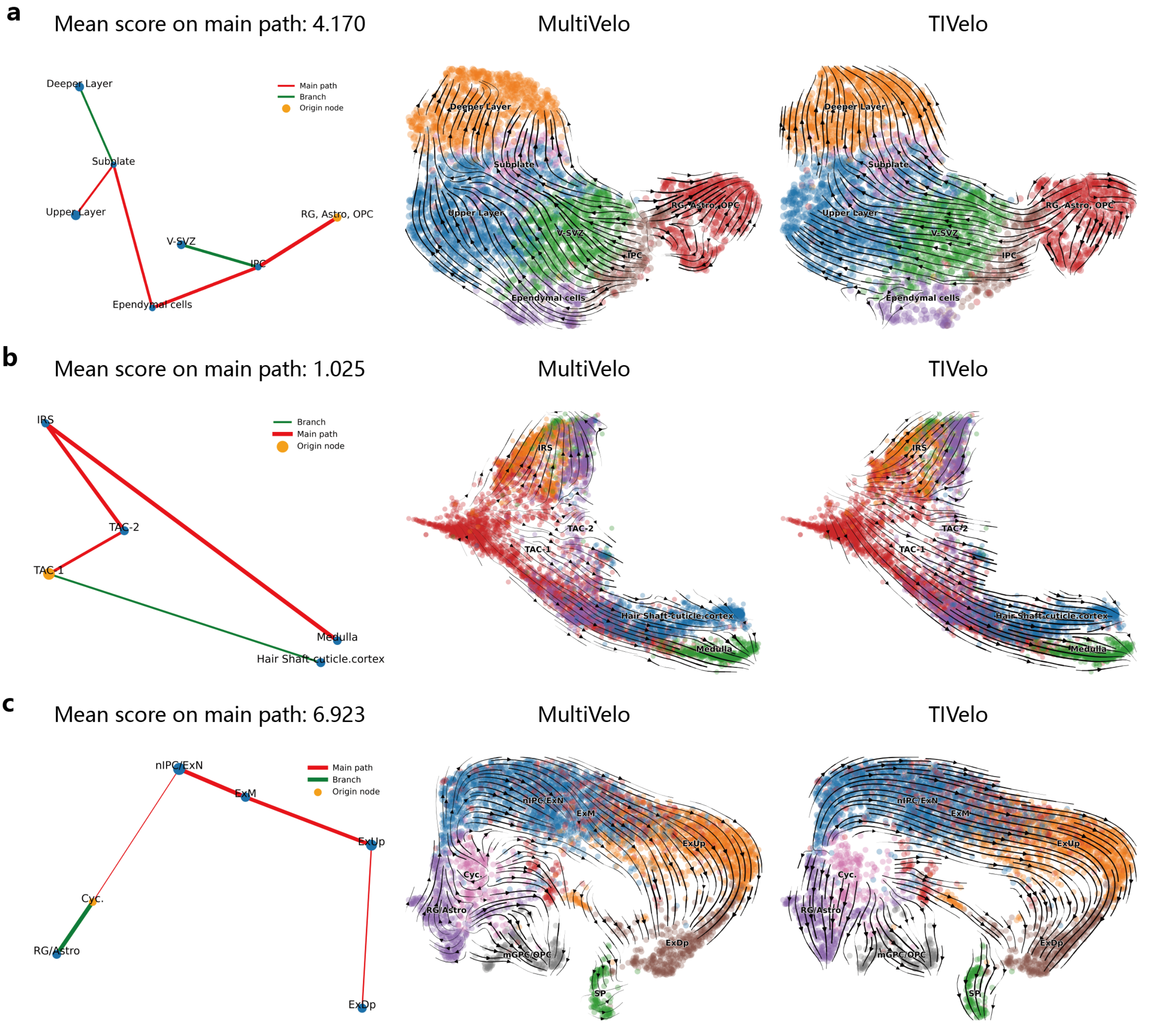

Supplementary Fig. 11 | Supplementary results on three single-cell multi-omics datasets. **a**. Left: The cluster graph after graph pruning and main path selection on embryonic mouse brain. Middle and right: Comparative velocity stream plots of embryonic mouse brain produced by MultiVelo and TIVelo. **b**. Left: The cluster graph after graph pruning and main path selection on SHARE-seq mouse skin. Middle and right: Comparative velocity stream plots of SHARE-seq mouse skin produced by MultiVelo and TIVelo. **c**. Left: The cluster graph after graph pruning and main path selection on developing human brain. Middle and right: Comparative velocity stream plots of developing human brain produced by MultiVelo and TIVelo.

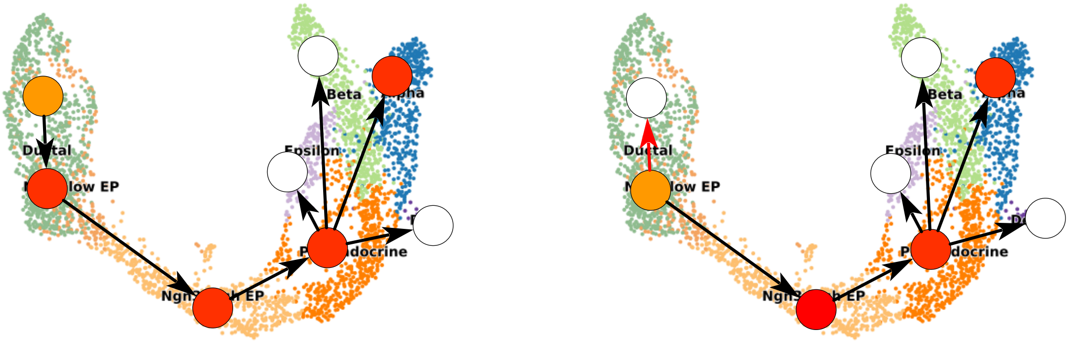

Supplementary Fig. 12 | The significance of selecting origin node in TIVelo. Left: If a root or end cluster is selected as the origin node (orange node), and the direction on the main path (orange & red nodes) is correctly inferred, the developmental direction of the entire graph can be correctly determined. Right: If an intermediate cluster is selected as the origin node, even if the main path direction is correct, some edges may still exhibit incorrect inferred directions (red arrow).

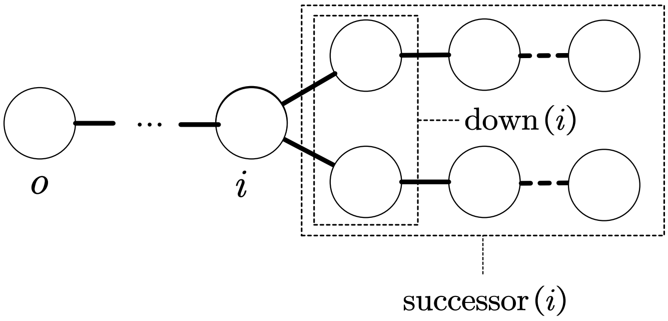

Supplementary Fig. 13 | Defining downstream and successor nodes. Downstream nodes for node i refers to nodes that directly connected to i, excluding any nodes located on the path from origin node o to node i. Successor nodes for node i refers to nodes that directly or indirectly connected to i, except those on the path between the origin node o and i.

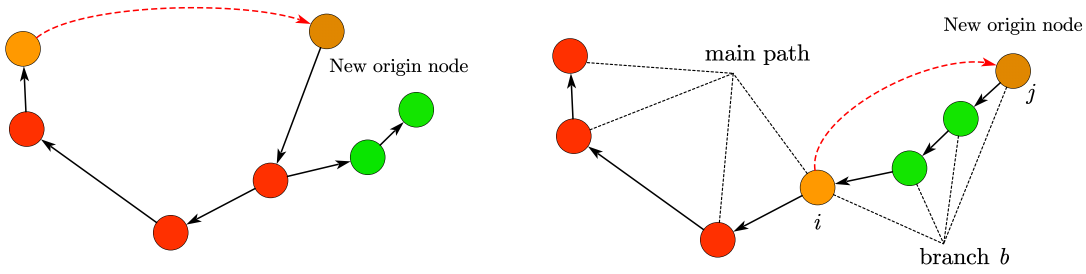

Supplementary Fig. 14 | Rules for inferring orientation and setting new origin node. Left: If $\frac{1}{G}\sum_g S_g < 0$, the current direction along the main path (orange & red nodes) is considered incorrect, and a new origin node should be set as the other end of the main path (orange node, shaded). Right: If an endpoint i of one branch b is also an endpoint of the main path, and the inferred direction on the main path is aligned with the inferred direction on branch b, the origin node (orange node) will be redefined as the other endpoint j of branch b (orange node, shaded).

Supplementary Notes

Note 1 The advantages of TIVelo over methods with variable kinetic rate parameters in the ODE equation

TIVelo leverages a model-free strategy based on an intrinsic property of the unspliced-spliced RNA relationship, to infer the velocity direction on the cluster level. Before TIVelo, some existing methods (e.g., DeepVelo[1], cellDancer[2]) have already accounted for variable kinetics rather than constant parameters. In this section, we expand our discussion comparing TIVelo with DeepVelo and cellDancer, highlighting key methodological differences and TIVelo's advantages.

DeepVelo

DeepVelo uses graph convolutional networks (GCN) to estimate cell-specific kinetic rates:

$$(\alpha, \beta, \gamma) = \mathrm{GCN}(U, S|A) \tag{1}$$

where $U, S \in \mathbb{R}^{N\times G}$ are cell by gene unspliced and spliced RNA input, $\alpha, \beta, \gamma \in \mathbb{R}^{N\times G}$ are estimated kinetic rates, and A is the adjacency matrix depicting the cell nearest neighbor graph.

The loss function designed by DeepVelo is as follows

$$\begin{aligned}
\mathcal{L}_+ &= \frac{1}{|\Omega|}\sum_{i\in\Omega}\left[s_i + \tilde{v}_i - \sum_{j\in\tilde{\mathcal{N}}_i} s_j P_{c+}(i\rightarrow j)\right]^2, \\
\mathcal{L}_- &= \frac{1}{|\Omega|}\sum_{i\in\Omega}\left[s_i - \tilde{v}_i - \sum_{j\in\tilde{\mathcal{N}}_i} s_j P_{c-}(i\leftarrow j)\right]^2, \\
\mathcal{L}_{\text{Pearson}} &= -\left(\lambda_u \operatorname{corr}(\tilde{v}_i, u_i) + \lambda_s \operatorname{corr}(\tilde{v}_i, -s_i)\right),
\end{aligned} \tag{2}$$

where Ω is the set of all cells, $\tilde{\mathcal{N}_i}$ is the estimated nearest neighborhood of cell i, and $\tilde{v}_i$ is the estimated velocity vector for the spliced RNA in cell i. $P_{c+}(i\rightarrow j)$ is the normalized binary indicator of cell i's neighboring cells j with $S_{\cos}(s_j - s_i, \tilde{v}_i) > 0$, indicating that cells j are the downstream cells of cell i according to the estimated velocity $\tilde{v}_i$. Similarly, $P_{c-}(i\rightarrow j)$ is the normalized binary indicator of cell i's neighboring cells j with $S_{\cos}(s_j - s_i, \tilde{v}_i) < 0$, indicating that cells j are the upstream cells of cell i according to $\tilde{v}_i$. These binary indicators are normalized across the neighborhood to form probability distributions.

It has been mentioned in the original DeepVelo paper[1] that the first two terms in Supplementary Equation (2) are symmetric to the sign of $\tilde{v}_i$, i.e., $\mathcal{L}_+(\tilde{v}_i) + \mathcal{L}_-(\tilde{v}_i) = \mathcal{L}_+(-\tilde{v}_i) + \mathcal{L}_-(-\tilde{v}_i)$. The sign of $\tilde{v}_i$ is determined by the third term, $\mathcal{L}_{\text{Pearson}}$. However, the design of $\mathcal{L}_{\text{Pearson}}$ may introduce bias to genes where cells are all in the induction/repression phase. Here we take the dataset mouse gastrulation (erythroid) as an example.

First, we compared the velocity stream plots inferred by DeepVelo and TIVelo. DeepVelo exhibits a backward velocity flow from Erythroid 3 to Erythroid 2, as shown in Supplementary Fig. 15(a) (blue box). This result was generated using DeepVelo's default parameter settings (`https://github.com/bowang-lab/DeepVelo/blob/main/README.md`). Notably, the result presented in Fig. S2 of the DeepVelo paper[1] is different and it required a specific training adjustment: the removal of $\mathcal{L}_{\text{Pearson}}$ from the loss function after 10 training epochs (`https://github.com/bowang-lab/DeepVelo/blob/main/examples/mouse_gastrulation.py`). This adjustment was not applied in the analysis of the other five datasets discussed in DeepVelo's paper: $\mathcal{L}_{\text{Pearson}}$ is retained throughout the training process.

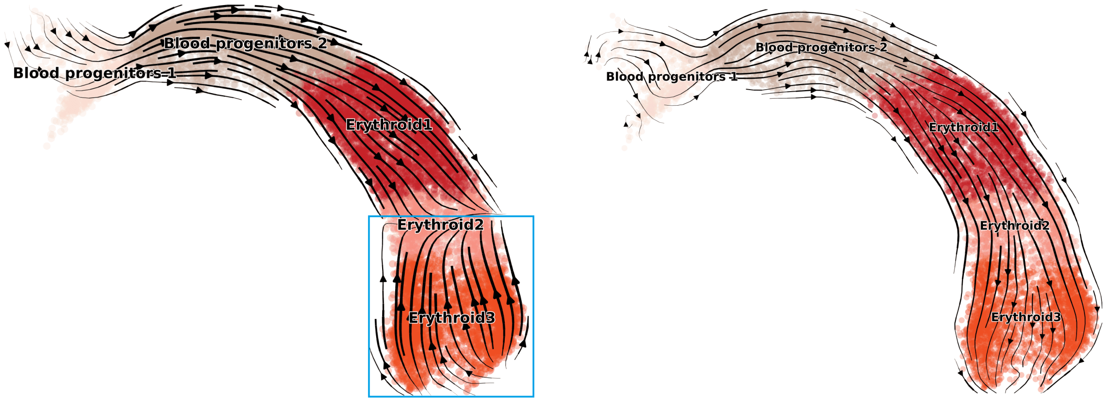

(a) DeepVelo velocity stream (b) TIVelo velocity stream

Supplementary Fig. 15 | The comparison of velocity stream plots inferred by DeepVelo and TIVelo in mouse gastrulation (erythroid). Blue box: backward velocity stream estimated by DeepVelo.

To further investigate the impact of including $\mathcal{L}_{\text{Pearson}}$ and why the default setting of DeepVelo fails in this dataset, we inspected three genes where all cells are in the repression phase, where the velocities for spliced RNA are expected to be negative for all cells. For each gene g, we visualized the fitted velocities $\tilde{v}_i^{(g)}$ for spliced RNA given by DeepVelo for a subset of sampled cells i. From the results shown in Supplementary Fig. 16, cells i with high u_i and low s_i tend to have positive $\tilde{v}_i^{(g)}$ (green box), while cells i with low u_i and high s_i tend to have negative $\tilde{v}_i^{(g)}$ (yellow box).

This behavior arises from the design of the $\mathcal{L}_{\text{Pearson}}$ term in the loss function (2). As a result of this term, cells with high u_i and low s_i tend to have positive velocities, and cells with low u_i and high s_i tend to have negative velocities, regardless of the transcriptional phase of cells. This is particularly problematic for genes with all cells entirely in the repression or induction phase, where velocities should be negative or positive across all cells, respectively. This inherent limitation in the design of $\mathcal{L}_{\text{Pearson}}$ in DeepVelo leads to the backward velocity stream from Erythroid 3 and Erythroid 2 observed in Supplementary Fig. 15(a) (blue box).

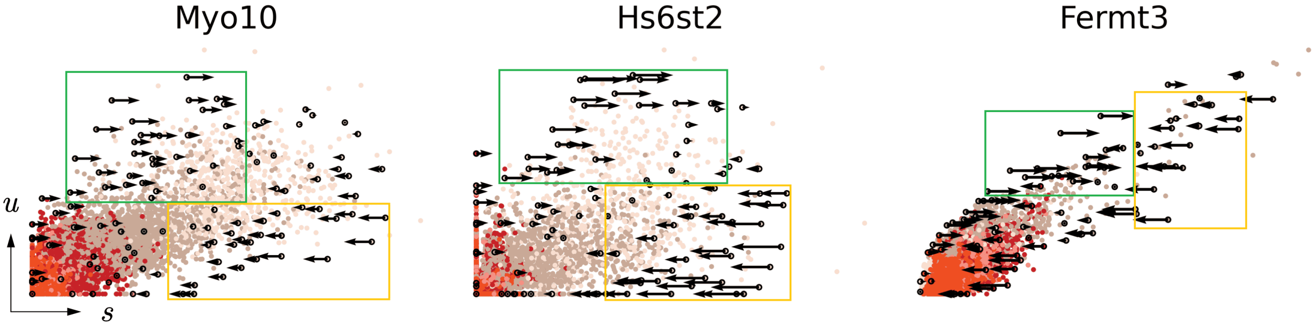

Supplementary Fig. 16 | The velocity inferred by DeepVelo for three genes in mouse gastrulation (erythroid). Here only the velocities for spliced RNA ($\tilde{v}_i$) are shown. Green box: cells with positive $\tilde{v}_i$ due to high u_i and low s_i. Yellow box: cells with negative $\tilde{v}_i$ due to low u_i and high s_i.

In contrast, TIVelo does not have the inherent constraints imposed by $\mathcal{L}_{\text{Pearson}}$ in DeepVelo, allowing genes with all cells in the repression (or induction) phase to exhibit negative (or positive) velocities across all cells, as shown in Supplementary Fig. 17.

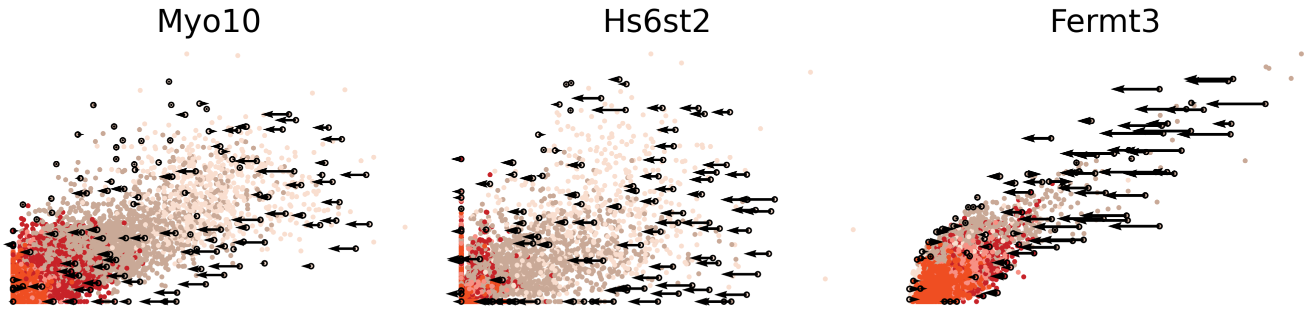

Supplementary Fig. 17 | The velocity inferred by TIVelo for three genes in mouse gastrulation (erythroid). Here only the velocities for spliced RNA ($\tilde{v}_i$) are shown.

cellDancer

cellDancer trains a deep neural network (DNN) Φ_{θ^g} for each gene g independently to estimate cell-specific kinetic rates $(\alpha_i^g, \beta_i^g, \gamma_i^g)$ for cell i:

$$(\alpha_i^g, \beta_i^g, \gamma_i^g)^{\mathrm{T}} = \Phi_{\theta^g}\left(u_i^g, s_i^g\right) \tag{3}$$

For each gene g, the loss function is designed as follows:

$$\begin{aligned} \mathcal{L} &= \sum_{i=1}^{n} \mathcal{L}_i \\ \mathcal{L}_i &= 1 - \max_{\{i'\}} \frac{v_i \cdot v_i'}{|v_i| \cdot |v_i'|} \end{aligned} \tag{4}$$

where $v_i = (v_i^u, v_i^s)$, $v_i^u = \alpha_i^g - \beta_i^g u_i^g$, $v_i^s = \beta_i^g u_i^g - \gamma_i^g s_i^g$. Cell i' is in the nearest neighborhood of cell i and $v_i' = (u_{i'}^g - u_i^g, s_{i'}^g - s_i^g)$.

For each gene, cellDancer minimizes $\mathcal{L}_i$ by selecting a cell i' in the nearest neighborhood of cell i. However, this neighboring cell i' can vary across different genes for the same cell i, potentially leading to errors in velocity direction estimation. This issue is illustrated using the dataset intestinal organoid.

First, we compared the velocity stream plots inferred by cellDancer and TIVelo. In intestinal organoid, the Stem cells differentiate into Goblet cells and Paneth cells in the secretory lineage, and differentiate into Enterocytes in the enterocyte lineage (Supplementary Fig. 18(a)). cellDancer's velocity estimation fails to accurately capture the differentiation trajectory from Stem cells into two distinct lineages, as shown in Supplementary Fig. 18(b) (red boxes).

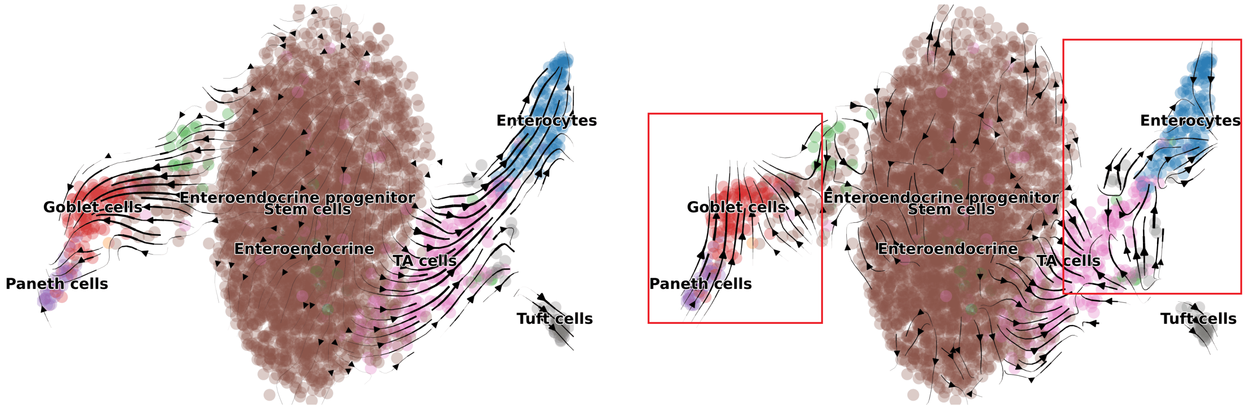

(a) TIVelo velocity stream

(b) cellDancer velocity stream

Supplementary Fig. 18 | The comparison of velocity stream plots inferred by TIVelo and cellDancer in intestinal organoid.

To further investigate this issue of cellDancer, we inspected six genes in this dataset, and visualized the velocities (v_i^u, v_i^s) estimated by cellDancer. For the first three genes, the estimated velocities indicate a reversed direction of differentiation from Enterocytes to Stem cells, as shown in Supplementary Fig. 19.

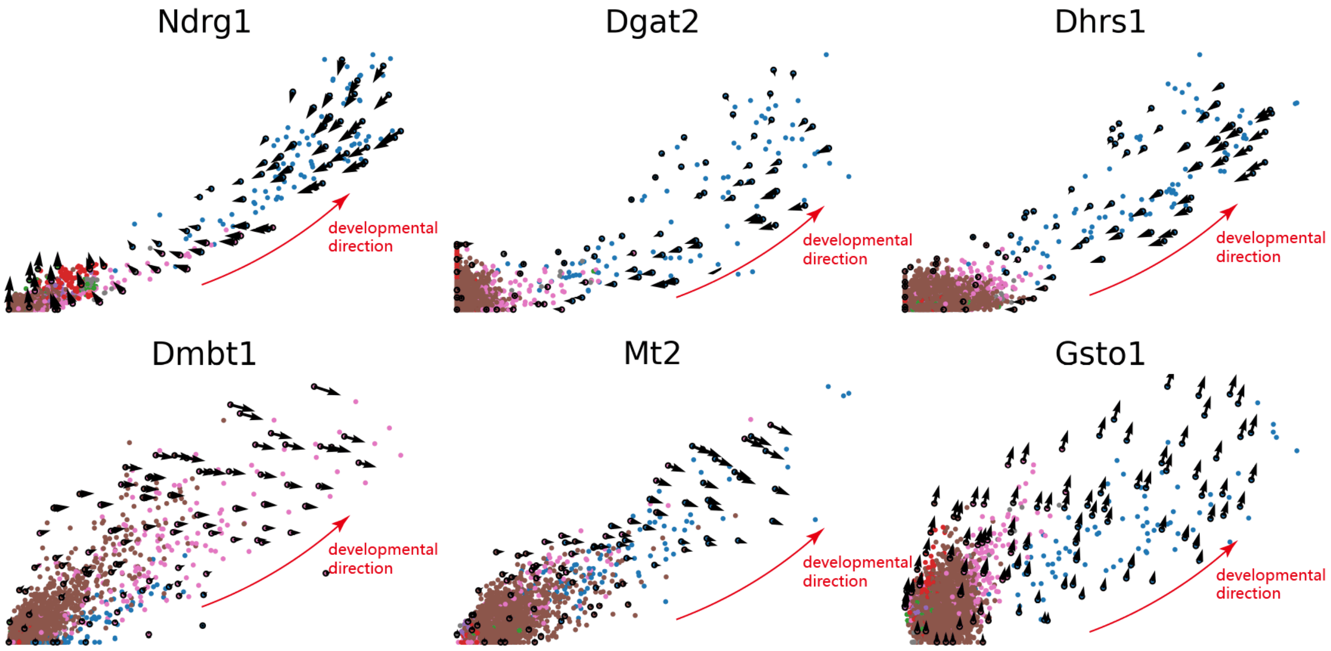

Supplementary Fig. 19 | The velocity inferred by cellDancer for six genes in intestinal organoid. Here both the velocities for unspliced RNA and for spliced RNA are shown.

We also inspected a randomly sampled subset of five cells and, for each cell i, identified its corresponding neighboring cell i' for each gene as defined in Supplementary Equation (4). In Supplementary Equation (4), the selected cell i' should be the downstream cells to cell i in differentiation. The results presented in Supplementary Fig. 20 reveal that for the first three genes, cellDancer tends to select the upstream cells of cell i as i', while for the last three genes, cellDancer tends to select cell i' from the downstream cells of cell i. The error in velocity estimation for such genes arises from the error in neighboring cell selection in cellDancer.

This error in the selection of neighboring cell i' for the top three genes arises from the flexible design of cellDancer's loss function (4), which does not guarantee that cell i' is selected from biologically plausible downstream cells of cell i. Consequently, the neighboring cell i' may be selected from the upstream cells of cell i, as demonstrated in Supplementary Fig. 20 (top row).

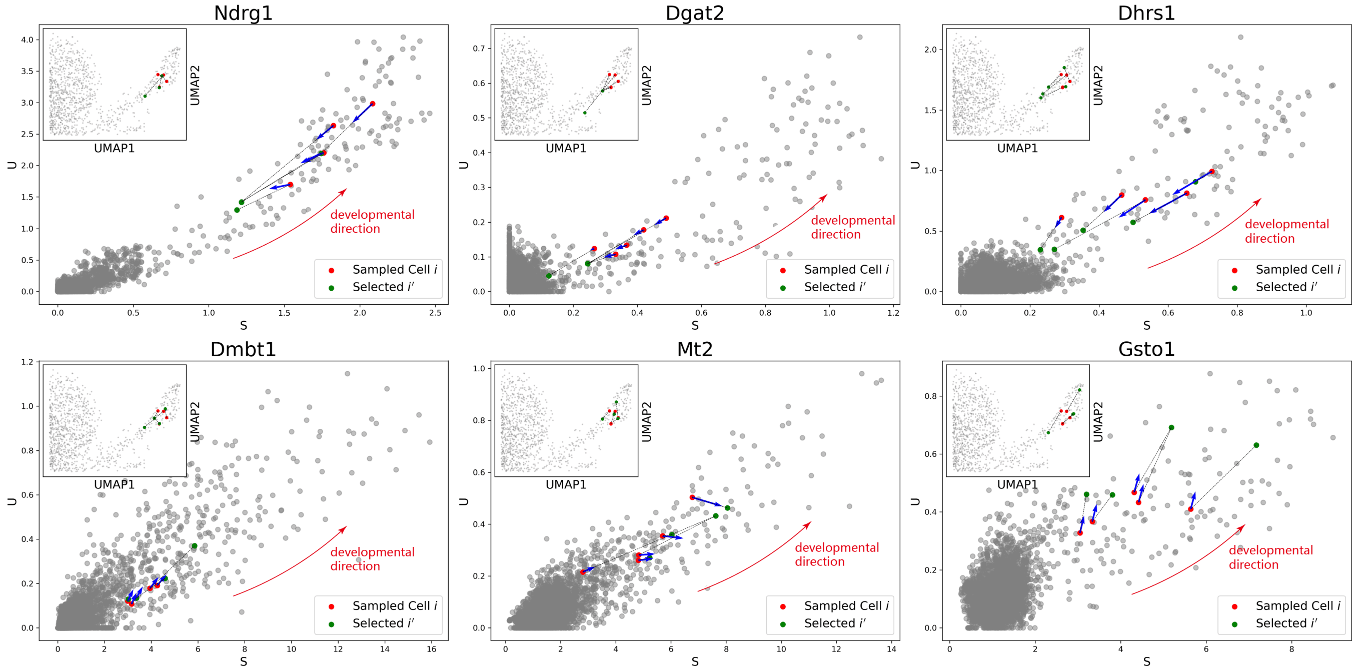

Supplementary Fig. 20 | Velocity estimation from cellDancer and the cells i' for five randomly sampled cells i. Red points: sampled cells i. Green points: the cells i' for corresponding cell i.

The design of L_i in Supplementary Equation (4) in cellDancer is too flexible with respect to the direction of v_i. Even if v_i is inverted or incorrect, we may still find a neighboring cell i' that makes L_i small. To further illustrate this limitation introduced by L_i in Supplementary Equation (4), we inverted the direction of inferred velocities from cellDancer for one randomly sampled cell i and a gene *Gsto1*, i.e., setting v_i to $-v_i$. We then selected cell i' for both v_i and $-v_i$, and compared the cosine similarity calculated in Supplementary Equation (4) based on v_i and $-v_i$. Notably, the cosine similarity for $-v_i$ is higher than that for v_i, leading to an even lower loss L_i compared to that with v_i, as shown in Supplementary Fig. 21. This demonstrates that the design of the loss L_i in cellDancer is too flexible, and regardless of the direction of v_i, there may exist a neighboring cell i' that makes L_i small.

In the contrary, in TIVelo, we selected the same directed nearest neighborhood $dnn(i)$ for each cell i across different genes (Supplementary Fig. 22), based on the overall direction on the main path. This strategy provides constraints on the inferred velocity of individual genes, ensuring that the velocity will point to the downstream cells, bypassing the limitation of L_i of Supplementary Equation (4) in cellDancer. In Supplementary Fig. 22, each blue arrow represents the RNA velocity for each selected cell and each gene, which is obtained based on the ensemble of neighboring cells in the dNN of the selected cell. In some cases, the velocity does not point towards the mean expression value of cells in the dNN, which is due to the regularization term in our loss objective to enhance the consistency of the velocity vector of similar cells (Methods).

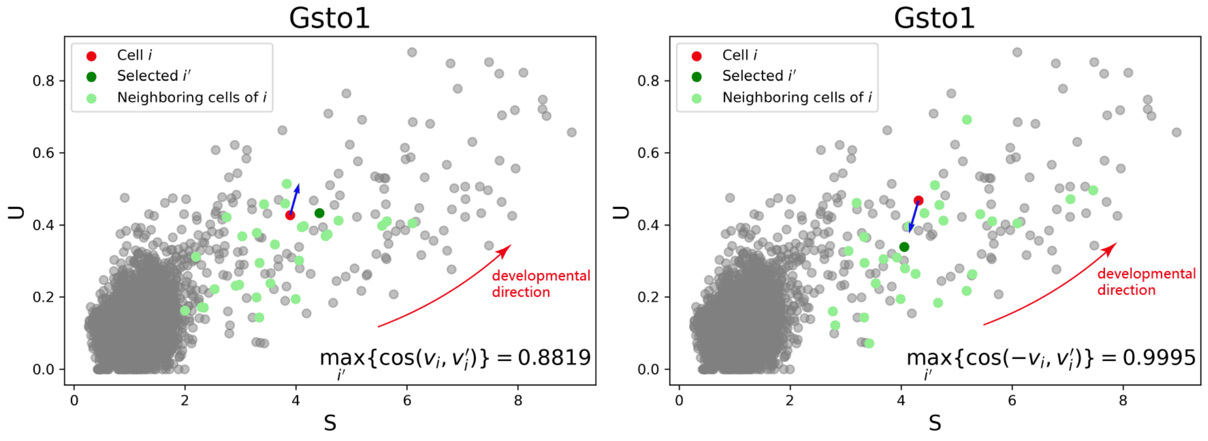

Supplementary Fig. 21 | Velocity estimation $v_i/-v_i$ and cell i' selected based on $v_i/-v_i$ for one randomly selected cell i. Red point: selected cell i. Green points: cell i' for cell i. Light green points: neighboring cells of cell i.

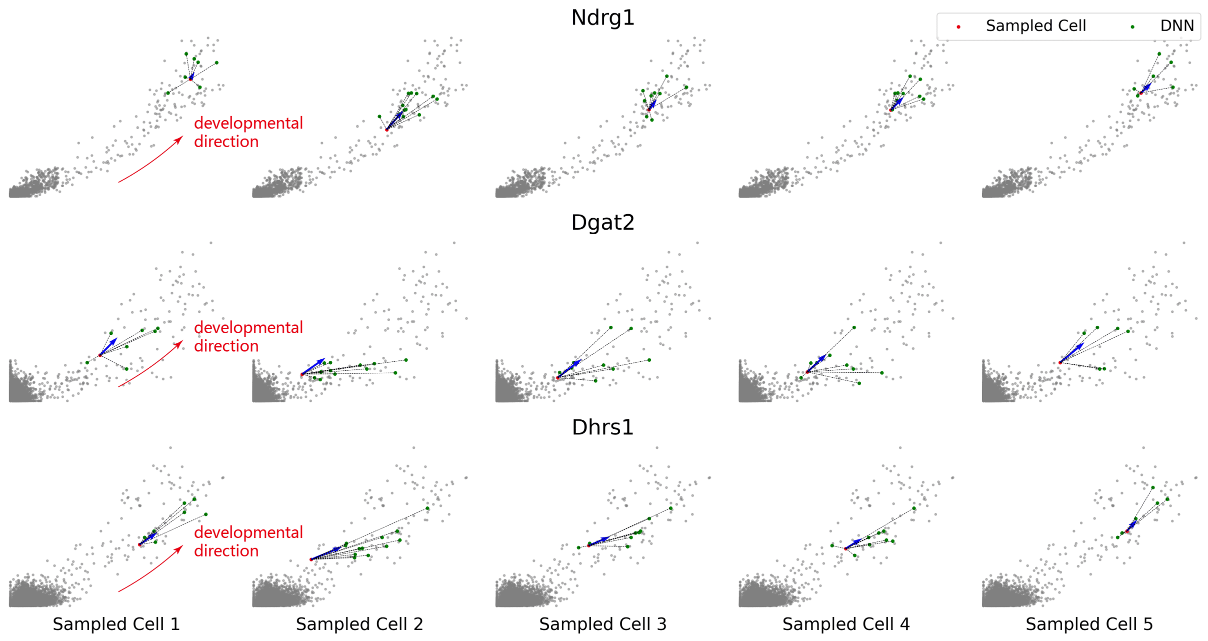

Supplementary Fig. 22 | Velocity estimation from TIVelo and the directed nearest neighborhood (dNN) for one randomly selected cell. Red point: selected cell i. Green points: dNN for cell i.

Note 2 Scientific findings based on TIVelo's inferred velocities

Fate Probability Visualization

To demonstrate how TIVelo's inferred velocities can contribute to biological studies, we present an analysis of the intestinal organoid dataset, highlighting key scientific insights enabled by TIVelo.

Fate probability analysis enables the quantification of each cell's likelihood to differentiate into specific terminal states. We calculated the fate probabilities of each cell in intestinal organoid based on TIVelo's inferred velocities[3]. These probabilities were visualized using circular projections[4] (Supplementary Fig. 23), where each terminal state was arranged at a vertex of an equilateral triangle, and cells are positioned inside the triangle according to their fate probabilities. The spatial proximity of a cell to a particular vertex directly reflects its tendency to differentiate towards the corresponding terminal state. This visualization provides a comprehensive overview of cell fate decisions during differentiation.

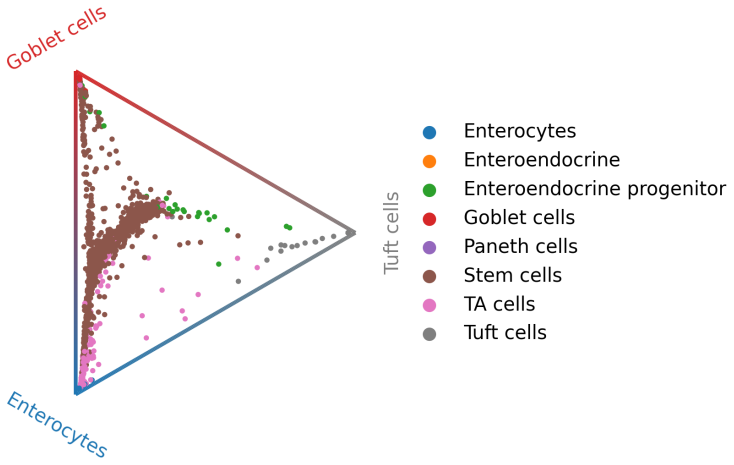

Supplementary Fig. 23 | Fate probabilities visualization towards terminal states via cirular projections[4], where each terminal state was arranged at a vertex of an equilateral triangle, and cells are positioned inside the triangle according to their fate probabilities.

Lineage-Specific Driver Gene Identification

The intestinal organoid dataset features two distinct lineages: the secretory lineage and the enterocyte lineage. Based on the velocities inferred by TIVelo, we identified driver genes associated with each lineage[3]. In Supplementary Fig. 24 and Supplementary Fig. 25, we show some candidate driver genes for both lineages, along with their expression patterns across all cells.

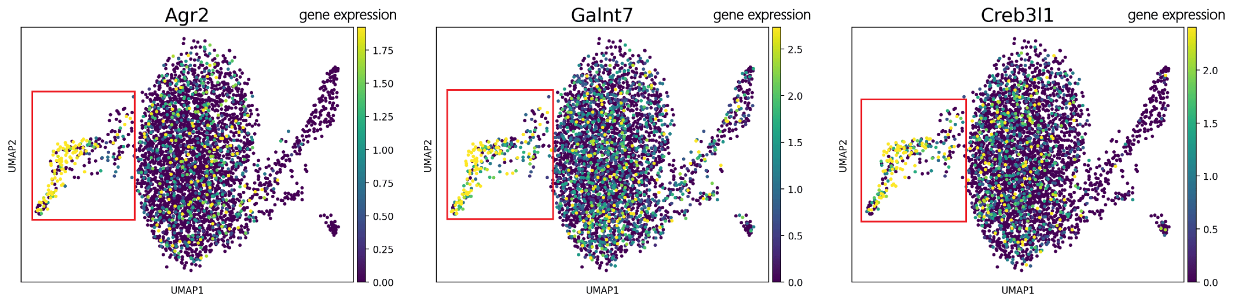

Supplementary Fig. 24 | Selected driver genes for secretory lineage (red boxes) and their expressions, identified based on TIVelo's inferred velocity.

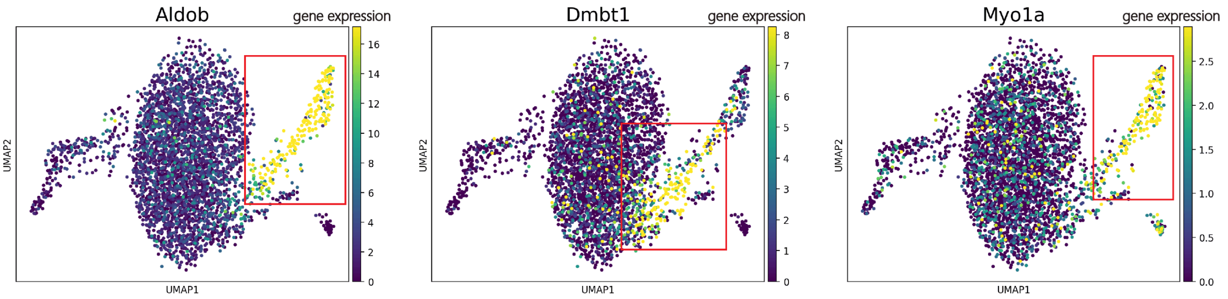

Supplementary Fig. 25 | Selected driver genes for enterocyte lineage (red boxes) and their expressions, identified based on TIVelo's inferred velocity.

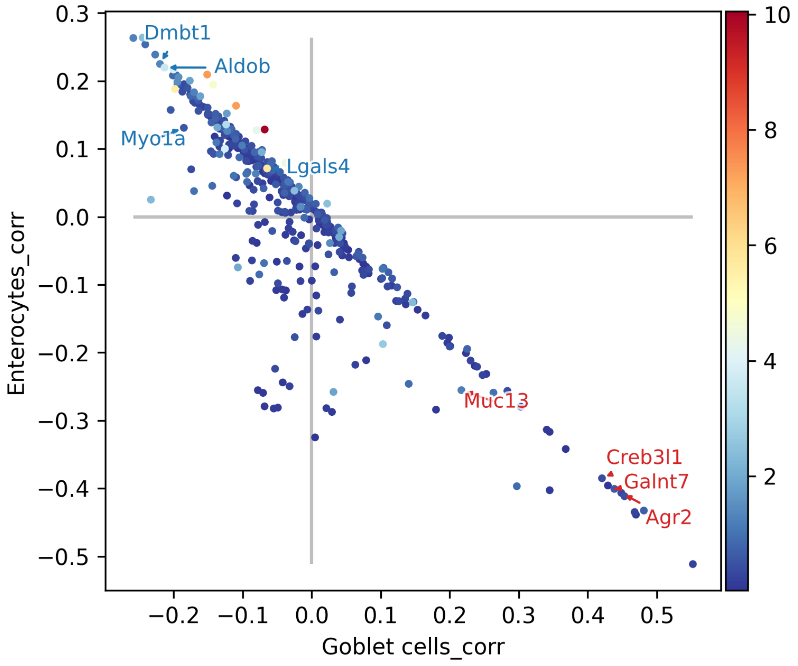

Supplementary Fig. 26 | The correlation of the gene expressions with fate probabilities (of two lineages) for genes in intestinal organoid.

Gene Name	Reference	Lineage
Agr2	Park et al., 2009[5]; Zhao et al., 2010[6]	Secretory
Galnt7	Bennett et al., 2012[7]	Secretory
Creb3l1	Fox et al., 2010[8]	Secretory
Muc13	Sheng et al., 2011[9]	Secretory
Aldob	Gao et al., 2023[10]	Enterocyte
Dmbt1	Kaemmerer et al., 2012[11]	Enterocyte
Myo1a	Mcconnell et al., 2007[12]	Enterocyte
Lgals4	Cao et al., 2016[13]	Enterocyte

Supplementary Table 1 | Some driver genes identified based on TIVelo's inferred velocity for both lineages, with references validating their functions for the corresponding lineage.

To further validate these findings, we analyzed the correlation between gene expressions and fate probabilities of each lineage (across cells) for genes in the data[3]. As shown in Supplementary Fig. 26, genes such as *Agr2*, *Galnt7*, *Creb3l1* and *Muc13* exhibit strong correlations with the secretory lineage, while genes like *Aldob*, *Dmbt1*, *Myo1a* and *Lgals4* are highly correlated with the enterocyte lineage. The functions of those identified driver genes for the corresponding lineage are supported by literature references, summarized in Supplementary Table 1.

Identification of Macrostates and Functional Insights

In the intestinal organoid dataset, we identified macrostates proposed in CellRank[14] to delineate metastable states within the Markov chain of cellular transitions. These macrostates represent cell populations that maintain relative stability during the differentiation process. Based on the velocities inferred by TIVelo[3], we identified a distinct macrostate within the "Stem cell" cluster, and subsequent pathway analysis revealed significant enrichment of the Ribosome pathway in this macrostate population.

Differential gene expression analysis of this macrostate compared to other stem cells revealed significant upregulation of ribosome-related genes (Supplementary Fig. 27(a)). KEGG pathway enrichment analysis further confirmed the enrichment of the Ribosome pathway (Supplementary Fig. 27(b)), suggesting that ribosome activity plays a critical role in intestinal stem cell (ISC) differentiation. This aligns with recent findings that ribosomes act as nutrient sensors, enabling ISCs to adapt to their local nutrient environment[15].

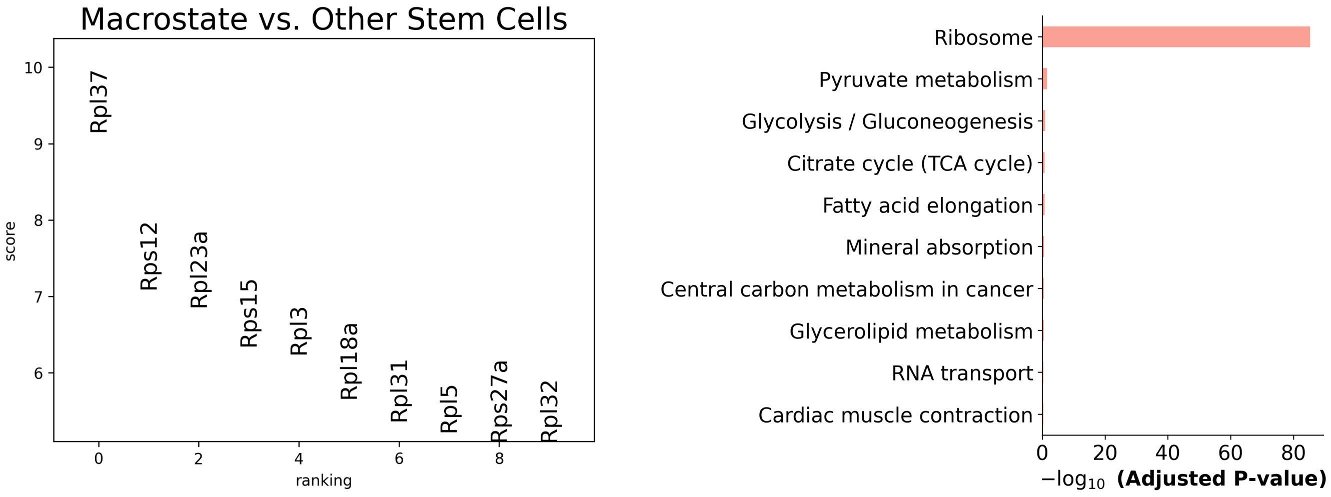

(a) DEG analysis

(b) KEGG analysis

Supplementary Fig. 27 | Left: differential gene expression analysis for macrostate we identified in "Stem cell" cluster against the rest stem cells. Right: KEGG analysis for differential expressed genes of macrostate we identified against the rest stem cells.

Kinetic Rates Inference by TIVelo

To provide more biological insights, we developed a kinetic rate mode for fitting RNA velocity in TIVelo, which enables simultaneous inference of cell-specific kinetic rates (α, β and γ) for each gene while maintaining the framework's core functionality (Methods). This reveals the cellular dynamics of the transcriptional phase for individual genes.

Supplementary Fig. 28 shows the comparison of velocity stream plots inferred by TIVelo and TIVelo (kinetic rate mode). The result inferred by TIVelo (kinetic rate mode) closely matches that from TIVelo. This indicates that TIVelo (kinetic rate mode) does not compromise velocity accuracy while providing additional biological information about the kinetic rates in cell dynamics.

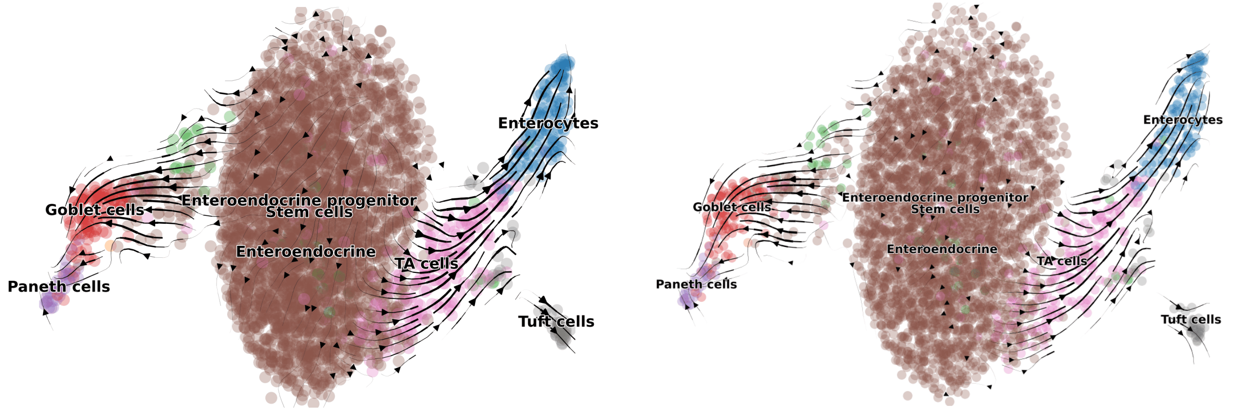

(a) TIVelo　　　(b) TIVelo (kinetic rate mode)

Supplementary Fig. 28 | The comparison of velocity stream plots inferred by TIVelo and TIVelo (kinetic rate mode) in intestinal organoid.

The inferred rate parameters α, β and γ reveal the transcriptional phase of individual genes, and we take two driver genes for enterocyte lineage in this dataset as examples. As shown in Supplementary Fig. 29, for genes *Aldob* and *Dmbt1*, there is a rapid increase of α and β rates for cells in the enterocyte lineage. This indicates that such cells are in the active transcription phases of their corresponding driver genes, providing additional biological insights for cell dynamics.

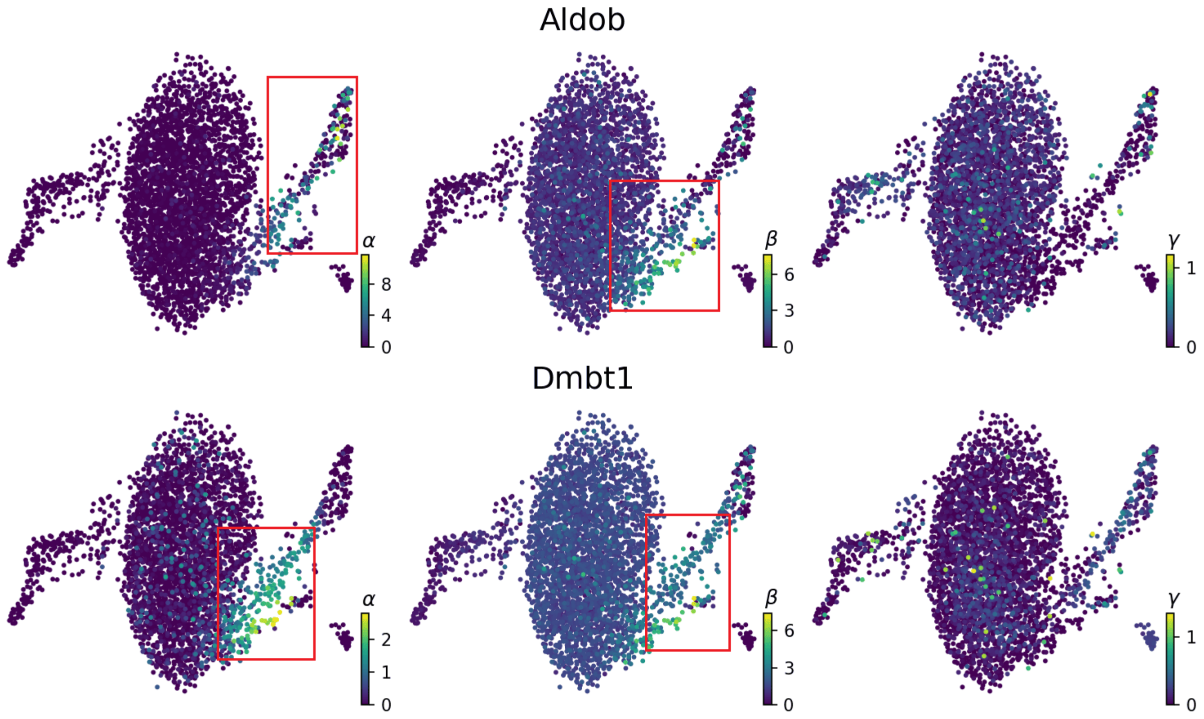

Supplementary Fig. 29 | Cell-specific kinetic rates α, β and γ inferred by TIVelo, for two driver genes of enterocyte lineage. Red boxes: the rapid growth of α and β for cells in the enterocyte lineage.

Note 3 TIVelo's inferred velocity for RPE1-FUCCI in cluster 1

For RPE1-FUCCI, the inferred velocity streams from TIVelo in the upper and lower regions of cluster 1 exhibit different orientations, which may initially appear inconsistent (Supplementary Fig. 30(a)). However, this directional pattern is not indicative of an inconsistency in TIVelo's velocity estimation but may rather reflect the underlying biological dynamics.

Firstly, cell cycle scores support the velocity stream of TIVelo in cluster 1. As demonstrated in Supplementary Fig. 30(b), which focuses specifically on cluster 1 cells with relatively large UMAP2 components, the cell cycle scores clearly indicate that cells in the upper region exhibit higher scores compared to those in the lower region. This supports the biological plausibility of the upward velocity direction observed in the upper region of cluster 1 (see Supplementary Fig. 30(a)).

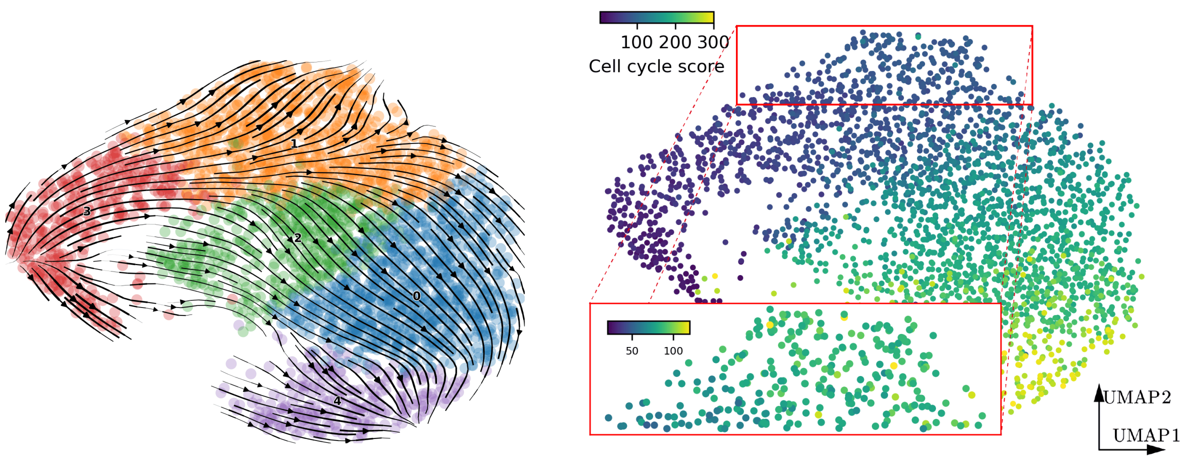

(a) (b)

Supplementary Fig. 30 | (a) Velocity stream plots for RPE1-FUCCI, inferred by TIVelo. (b) Cell cycle scores of RPE1-FUCCI cells, highlighting cluster 1 cells with relatively large UMAP2 components.

Secondly, similar directional patterns in cluster 1 are also observed in the velocity stream plots inferred by UniTVelo and DeepVelo, as demonstrated in Supplementary Fig. 31.

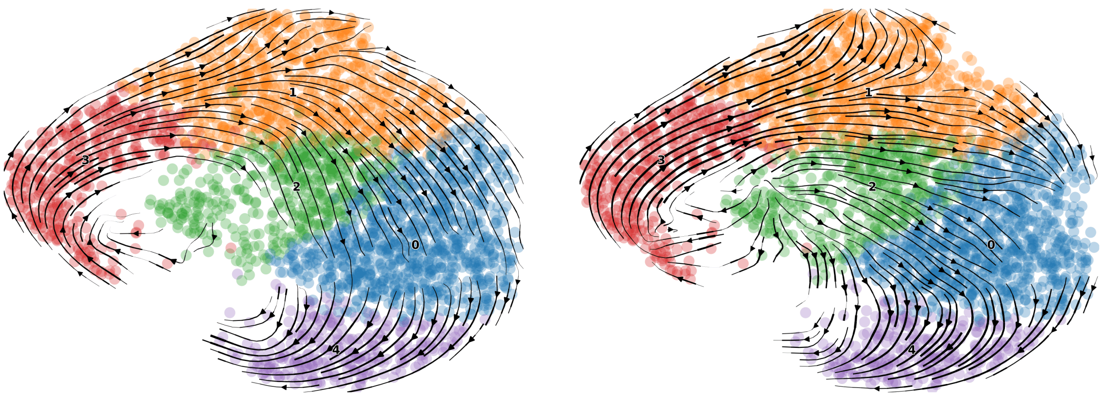

(a) UniTVelo (b) DeepVelo

Supplementary Fig. 31 | Velocity stream plots for RPE1-FUCCI, inferred by UniTVelo and DeepVelo.

In addition, while the directional pattern in cluster 1 is less pronounced in the velocity stream plot

of veloVI[16] (Supplementary Fig. 32(a)), plotting the velocity vectors longer reveals a similar trend (Supplementary Fig. 32(b)).

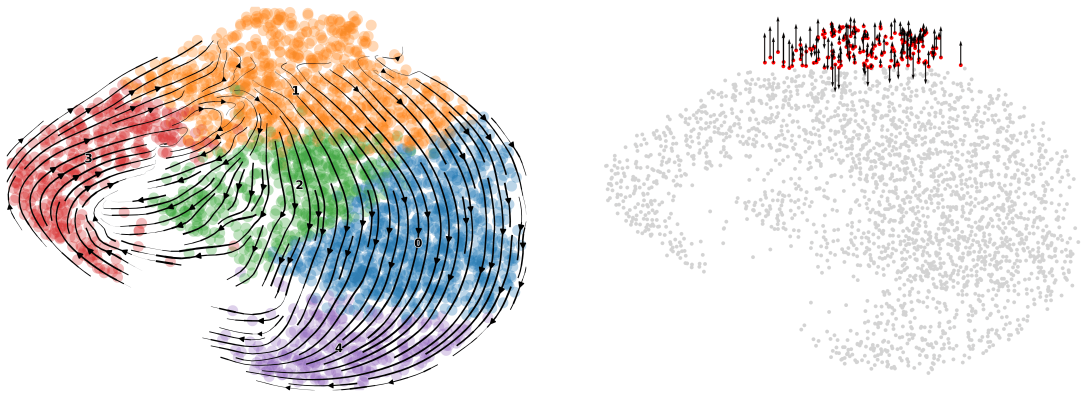

(a) (b)

Supplementary Fig. 32 | (a) Velocity stream plots for RPE1-FUCCI, inferred by veloVI. (b) Inferred RNA velocity vectors from veloVI projected onto 2D UMAP. Only the second velocity component for cells with relatively large UMAP2 components (highlighted in red) is shown. The length of the velocity vectors has been scaled up for clarity.

Note 4 Origin node selection of TIVelo

In our framework, terminal states (root cluster or end clusters) are identified using `scvelo.tl.terminal_states`[17]. The origin node is selected as the cell cluster exhibiting the strongest signal as the root cluster or the end cluster among the inferred terminal states. In detail, the procedure for the origin node selection in TIVelo involves the following steps:

Firstly, `scvelo.tl.terminal_states` calculates the transition matrix $\pi_{c,c'} = \cos\angle(\delta_{c,c'}, v_c)$, where v_c is the inferred velocity of cell c, $\delta_{c,c'} = s_{c'} - s_c$ and s_c is the spliced expression of cell c. $\pi_{c,c'}$ is normalized by

$$\tilde{\pi}_{c,c'} = \frac{1}{z_c} \exp(\frac{\pi_{c,c'}}{\sigma_c^2}) \tag{5}$$

with row normalization factors $z_c = \sum_{c'} \exp(\frac{\pi_{c,c'}}{\sigma_c^2})$ and kernel width parameters σ_c optionally adjusted for each cell locally.

Secondly, the end and root clusters are obtained as stationary states of the velocity-inferred transition matrix $\tilde{\pi}$ and its transpose $\tilde{\pi}^{\mathrm{T}}$, respectively. A root score vector μ^{root} and an end score vector μ^{end} will be calculated for all cells, which is given by left eigenvectors corresponding to an eigenvalue of 1, that is

$$\mu^{\text{end}} = \mu^{\text{end}}\,\tilde{\pi}, \quad \mu^{\text{root}} = \mu^{\text{root}}\tilde{\pi}^{\mathrm{T}}. \tag{6}$$

An example of inferred μ^{root} and μ^{end} by scVelo for dataset HSPCs is shown in Supplementary Fig. 33.

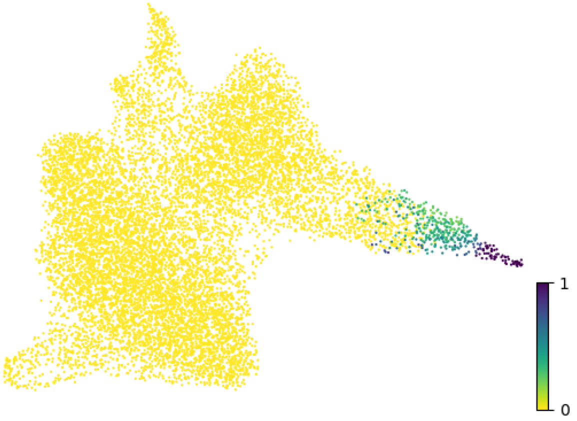

(a) Root score

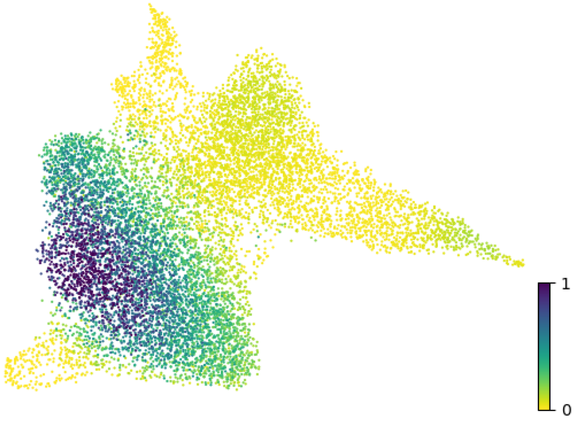

(b) End score

Supplementary Fig. 33 | Root score (μ^{root}) and end score (μ^{end}) for HSPCs inferred by scVelo.

Finally, the cell cluster with the largest mean root score will be selected as the origin node. If there is no cell cluster with a root score larger than a threshold (0.1), we select the one with the largest mean end score as the origin node (Methods).

For multi-branch datasets with multiple expected end clusters, this strategy for selecting the origin node remains effective, as long as one of the end clusters or the root cluster is selected as the origin node. We illustrate this using the intestinal organoid dataset with two lineages.

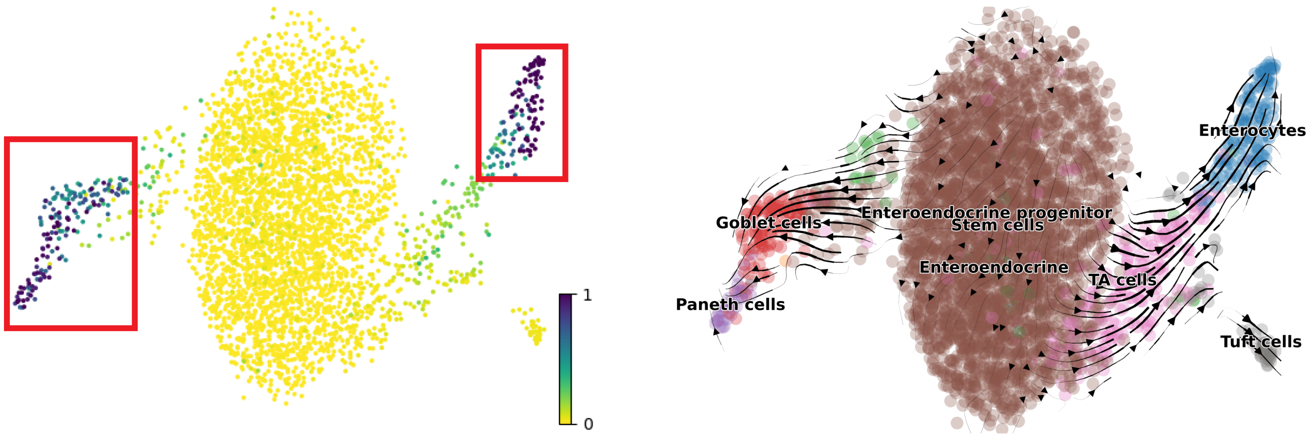

(a) (b)

Supplementary Fig. 34 | (a) Root score (μ^{root}) of intestinal organoid inferred by scVelo. (b) Velocity stream plot inferred by TIVelo, based on either Enterocytes or Paneth cell selected as the origin node.

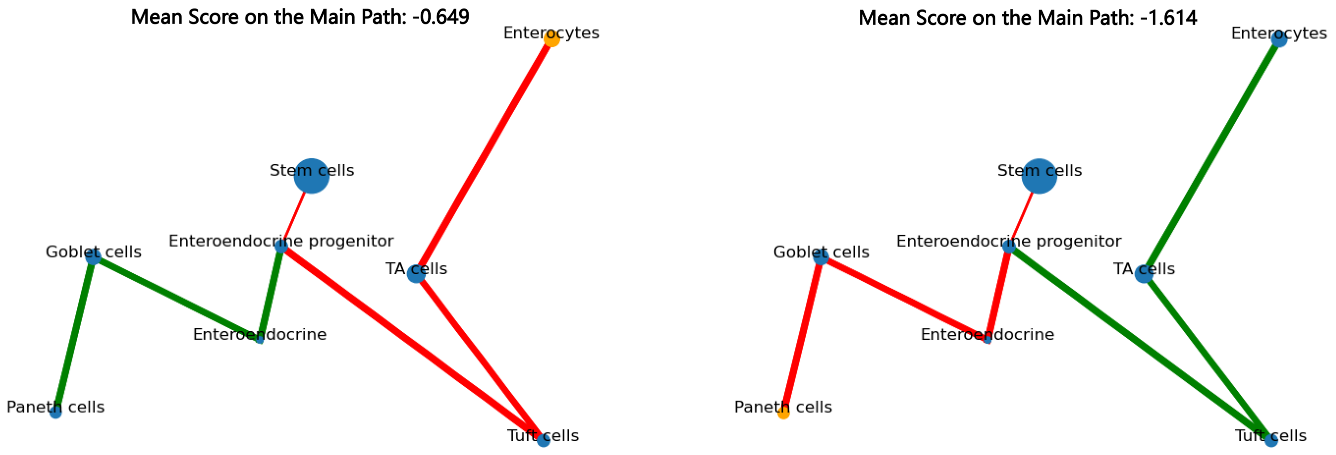

(a) Main path selection 1 (b) Main path selection 2

Supplementary Fig. 35 | The main path selection in intestinal organoid. Main path selection 1 is based on the case when Enterocytes is selected as the origin node, while main path selection 2 is based on the case when Paneth cells is selected as the origin node.

As shown in Supplementary Fig. 34(a), the origin node is selected based on the root score. In this dataset, two cell clusters (Enterocytes and Paneth cells) exhibit relatively high root scores (red boxes). Regardless of whether the Enterocytes or Paneth cell cluster is selected as the origin node, TIVelo produces the same (and correct) velocity inference (Supplementary Fig. 34(b)). Depending on whether the Enterocytes or Paneth cell is selected as the origin node, different main paths are inferred, as demonstrated in Supplementary Fig. 35(a) and (b). The mean orientation scores inferred on both main paths are negative, and TIVelo correctly infers the velocity direction along both main paths.

`scvelo.tl.terminal_states` does not directly select cell clusters based on extreme u and s values. Nevertheless, we observed that the origin node selected by `scvelo.tl.terminal_states` often exhibits extreme values of unspliced and spliced RNA. We performed the following analysis for several multi-branch datasets, to demonstrate that the origin node selected exhibits a relatively high or low expression level of spliced RNA in the data:

1. For each gene g, we calculated the mean expression of s for each cell cluster i, denoted as $s_{i,g}$;
2. For each gene g and each cell cluster i, we calculated the relative expression as $\tilde{s}_{i,g} = s_{i,g}/s_{g,\max}$, where $s_{g,\max}$ are the maximum expression values of s in gene g.
3. We visualized the distribution of $\tilde{s}_{i,g}$ using violin plots for each cell cluster i.

The results shown in Supplementary Fig. 36 confirm that origin nodes typically exhibit extreme (high or low) relative s expression levels compared to other cell clusters.

Consistent with the theory of transcriptional kinetics, terminal states (root or end clusters) typically occupy the initiation or termination phases of transcriptional processes for most genes. Therefore, these states tend to exhibit extreme relative expression levels of s, which can be easily identified using `scvelo.tl.terminal_states`.

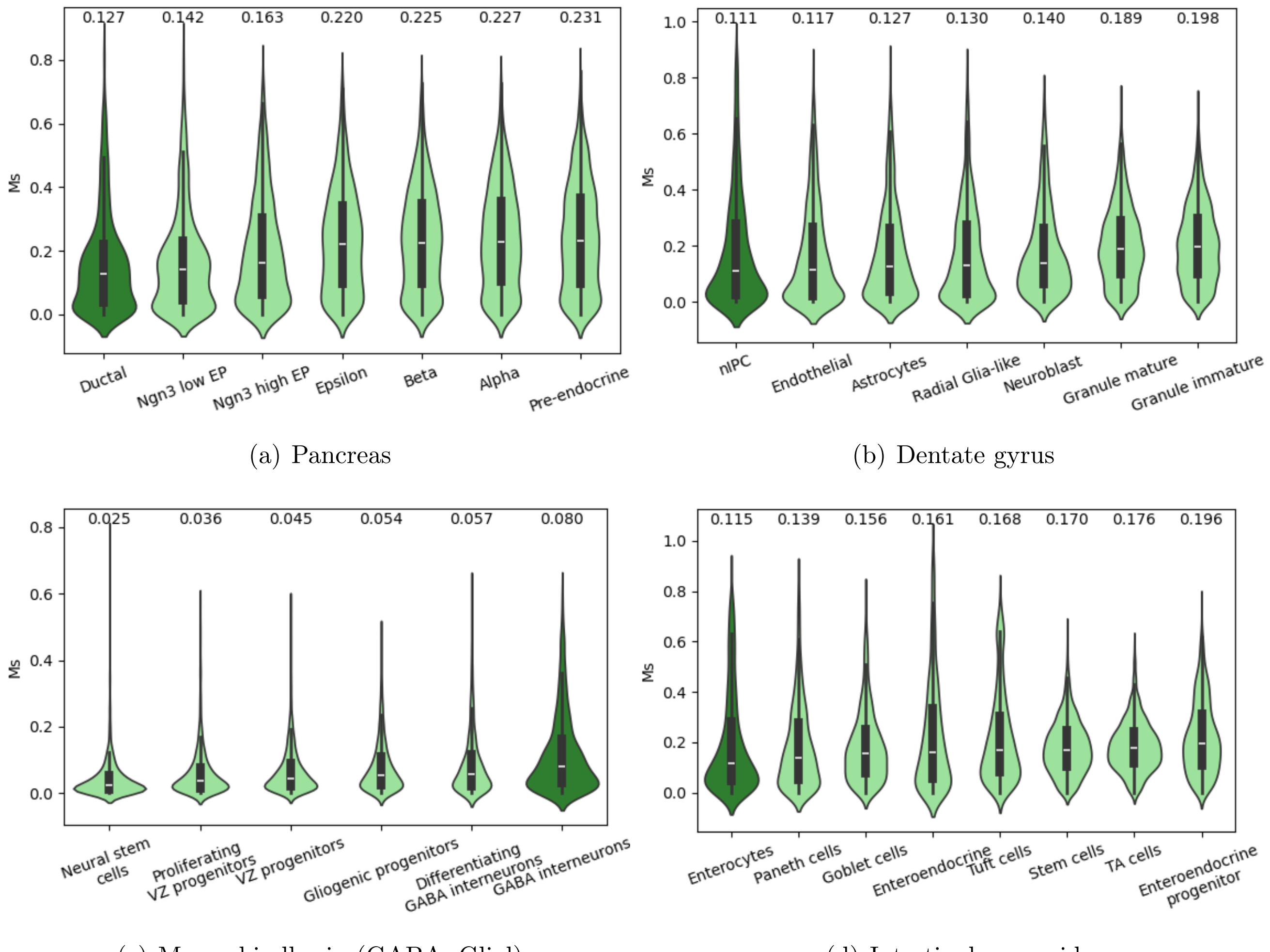

(a) Pancreas

(b) Dentate gyrus

(c) Mouse hindbrain (GABA, Glial)

(d) Intestinal organoid

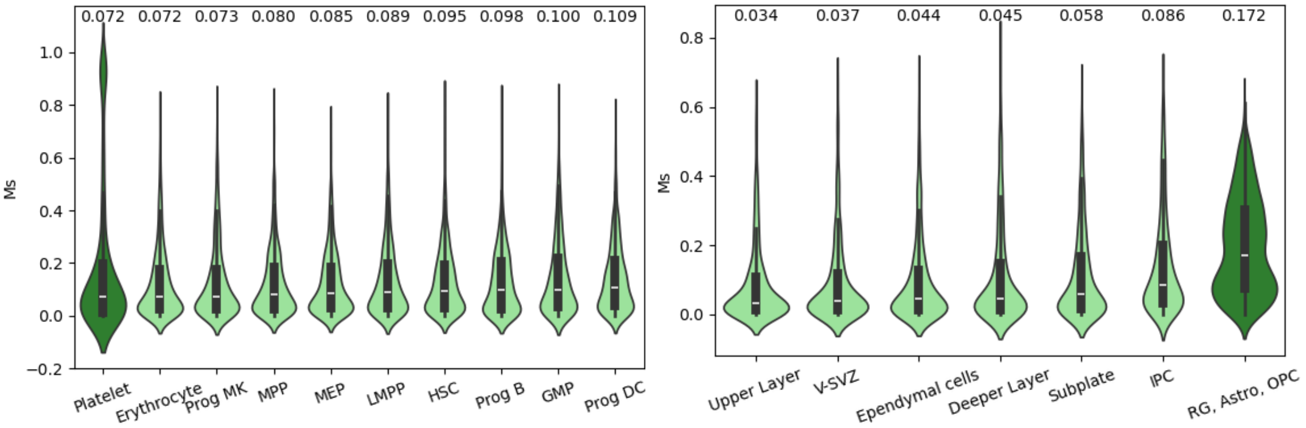

(e) HSPCs

(f) Mouse brain

Supplementary Fig. 36 | The violin plot of relative expression of s for each cell cluster in several multi-branch datasets. The violin for the origin node is marked by darker color. In the box plot inside each violin, the lower bound, centre and upper bound of the box plot stand for the first quartile (Q_1), median and the third quartile (Q_3) of the $\tilde{s}_{i,g}$, respectively. The lower whisker and the upper whisker stand for $Q_1 - 1.5 \times \text{IQR}$ and $Q_3 + 1.5 \times \text{IQR}$, where $\text{IQR} = Q_3 - Q_1$. The median values of each violin are annotated.

Note 5 Normalization in orientation score calculation

In orientation score calculation, u and s should be normalized before constructing the linear tree. Specifically, u and s are rescaled to sum to 1 for each gene. An alternative normalization approach is to normalize by the maximum values of u and s for each gene, i.e., $\tilde{u}_g = u_g/u_{g,\max}$ and $\tilde{s}_g = s_g/s_{g,\max}$. We compared the performance of both normalization approaches across 16 datasets, to see if the velocity direction on the main path can be correctly inferred. As shown in Supplementary Table 2, this alternative way of normalization cannot correctly infer the velocity direction on the main path in four datasets (intestinal organoid, mouse hindbrain (GABA, Glial), RPE1-FUCCI and U2OS-FUCCI). Therefore, we use the strategy of normalization by sum as the default approach.

Datasets	Sum	Direction	Maximum	Direction
Pancreas	16.072	✓	7.915	✓
Dentate gyrus	1.138	✓	2.311	✓
Gastrulation	4.742	✓	0.507	✓
Hindbrain (Oligo)	48.319	✓	14.965	✓
Intestinal organoid	-0.649	✓	2.919	✗
Retina	-6.399	✓	-2.063	✓
scNT	-17.159	✓	-9.801	✓
Dentate gyrus 2	9.082	✓	3.033	✓
Reprogramming	1.401	✓	1.210	✓
Hindbrain (GABA, Glial)	-0.158	✓	1.515	✗
RPE1-FUCCI	7.965	✓	-2.443	✗
U2OS-FUCCI	-1.610	✓	6.085	✗
Mouse brain	0.933	✓	1.129	✓
Mouse skin	1.025	✓	2.099	✓
HSPCs	-7.773	✓	-1.703	✓
Human brain	6.923	✓	7.066	✓

Supplementary Table 2 | The comparison of inferred velocity direction on the main path, by two normalization ways across the 16 datasets. Sum: mean orientation score (across all genes) on the main path, by normalization in which u and s are rescaled to sum to 1. Maximum: mean orientation score (across all cells and genes) on the main path, by normalization in which u and s are rescaled by $u_{g,\max}$ and $s_{g,\max}$. Direction: if the inferred velocity direction on the main path is correct.

Note 6 Robustness to the main path selection

Firstly, to evaluate how robust TIVelo is in correcting the directionality and the choice of the main path, we added perturbations to the original main path M selected by TIVelo. For datasets containing multiple branches, the perturbed main path incorporates one new branch that was not included in the original main path, while for single-branch datasets, the perturbed main path is shorter compared to the original main path. The details for obtaining the perturbed main path M' are as follows:

Denote the main path as $M = \{O, C_1, \cdots, C_m\}$, where O is the origin node and $C_1, \cdots, C_m$ are subsequent nodes.

1. For the case when the expected root cluster is selected as the origin node:
   - If there is one branch B connecting to C_j, $j = 1, \cdots, m$, namely $B = \{C_j, C'_1, \cdots, C'_n\}$, then we select a perturbed main path $M' = \{O, C_1, \cdots, C_j, C'_1, \cdots, C'_n\}$.
   - If there are several such branches $B_1, \cdots, B_b$, we prioritize the branch that maximizes the number of cells included in the perturbed main path.
   - If there is no such branch, we drop the last cluster C_m in the main path, and the perturbed main path is selected as $M' = \{O, C_1, \cdots, C_{m-1}\}$.
2. For the case when the expected end cluster is selected as the origin node:
   - If there exist several expected end clusters, we select another expected end cluster as the origin node O'. The perturbed main path is selected based on the new origin node O' (Methods).
   - If there is only one expected end cluster, we drop O in the main path, and the perturbed main path is selected as $M' = \{C_1, \cdots, C_{m-1}\}$.

For the perturbed main path, we tested whether TIVelo can correctly infer the expected direction on the main path. The results are summarized in Supplementary Table 3.

Datasets	Score on M	Direction	Score on M'	Direction
Pancreas	16.072	✓	16.371	✓
Dentate gyrus	1.138	✓	3.905	✓
Gastrulation	4.742	✓	5.106	✓
Hindbrain (Oligo)	48.319	✓	48.570	✓
Intestinal organoid	-0.649	✓	-1.614	✓
Retina	-6.399	✓	-0.006	✓
scNT	-17.159	✓	-13.269	✓
Dentate gyrus 2	9.082	✓	4.640	✓
Reprogramming	1.401	✓	10.188	✗
Hindbrain (GABA, Glial)	-0.158	✓	-1.292	✓
RPE1-FUCCI	7.965	✓	9.906	✓
U2OS-FUCCI	-1.610	✓	-1.544	✓
Mouse brain	0.933	✓	5.215	✓
Mouse skin	1.025	✓	-1.464	✗
HSPCs	-7.773	✓	0.093	✗
Human brain	6.923	✓	4.163	✓

Supplementary Table 3 | The comparison of velocity direction inferred by TIVelo for the original main path M and the perturbed main path M' across the 16 datasets. Score on $M(M')$: mean orientation score on the main path $M(M')$. Direction: if the inferred velocity direction on the main path $M(M')$ is correct.

For all 16 datasets used in TIVelo, the inferred velocity direction of the perturbed main path M' from TIVelo remains correct in 13 of them. This indicates that the velocity direction of the main path inferred by TIVelo is robust to moderate perturbations to the main path.

Secondly, for datasets where the mean orientation score on the main path M is positive (there are ten datasets in total), we reversed the direction of the main path, i.e., main path $M = \{O, C_1, \cdots, C_m\}$ will become $M' = \{C_m, \cdots, C_1, O\}$. We then assessed if TIVelo can correctly infer the velocity direction of the perturbed main path M'. The results of this evaluation are summarized in Supplementary Table 4.

From the analysis, TIVelo can correctly infer the velocity direction of the new main path M' in eight out of ten datasets we tested. This finding further underscores the robustness of TIVelo in handling variations in main path selection.

Datasets	Score on M	Direction	Score on M'	Direction
Pancreas	16.072	✓	-15.501	✓
Dentate gyrus	1.138	✓	0.184	✗
Gastrulation	4.742	✓	-13.490	✓
Hindbrain (Oligo)	48.319	✓	-50.613	✓
Dentate gyrus 2	9.082	✓	0.189	✓
Reprogramming	1.401	✓	2.119	✗
RPE1-FUCCI	7.965	✓	-19.005	✓
Mouse brain	0.933	✓	-3.637	✓
Mouse skin	1.025	✓	-1.968	✓
Human brain	6.923	✓	-3.166	✓

Supplementary Table 4 | The comparison of velocity direction inferred by TIVelo for the original main path M and the perturbed main path M' across ten datasets where the mean orientation score on the main path M is positive. Score on $M(M')$: mean orientation score on the main path $M(M')$. Direction: if the inferred velocity direction on the main path $M(M')$ is correct.

Note 7 Robustness to the origin node selection

The selection of the origin node impacts the main path selection and more specifically the inference of root node in TIVelo. To evaluate this impact of the different selections of origin node on TIVelo's performance, in each dataset we selected the terminal state O' with the second strongest signal as the origin node. The details for selecting this new origin node and its corresponding main path are as follows:

1. If the origin node O is selected according to the root score (i.e., with largest R_O and $R_O > 0.1$);
   - A new origin node O' is selected when the end score $E_{O'}$ is the largest and $E_{O'} > 0.1$;
   - If there is no such O', O' is selected as the cell cluster with the second largest root score.
2. If the origin node O is selected according to the end score (i.e., with largest E_O and $E_O > 0.1$);
   - A new origin node O' is selected when the root score $R_{O'}$ is the largest and $R_{O'} > 0.1$;
   - If there is no such O', O' is selected as the cell cluster with the second largest end score.
3. The procedure for selecting main path remains the same as before using the new origin node (Methods).

For the newly selected origin node O' and its corresponding main path M', we tested whether TIVelo can correctly infer the expected root cluster R: Specifically, $M' = \{O', C_1, \cdots, C_m\}$ and we tested if $O' = R$ when the mean orientation score on M' is positive, and if $C_m = R$ when the mean orientation score on M' is negative. If the root cluster is inferred correctly in TIVelo, it is likely that the velocity inference will also be accurate. The results are summarized as follows.

Datasets	O	Root	O'	Root
Pancreas	Ductal	✓	Beta	✓
Dentate gyrus	nIPC	✓	Astrocytes	✗
Gastrulation	Blood pro-genitors 1	✓	Erythoid 2	✓
Hindbrain (Oligo)	OPCs	✓	MFOLs	✓
Intestinal organoid	Paneth cells	✓	Entrocytes	✓
Retina	AC/HC	✓	PR	✓
scNT	Time 120	✓	Time 60	✓
Dentate gyrus 2	nIPC	✓	Granule	✗
Reprogramming	Cluster 9	✓	Cluster 3	✗
Hindbrain (GABA, Glial)	GABA in-terneurons	✓	Differentiat-ing GABA	✓
RPE1-FUCCI	Leiden 3	✓	Leiden 1	✗
U2OS-FUCCI	Leiden 1	✓	Leiden 2	✓
Mouse brain	RG, Astro, OPC	✓	Deeper Layer	✗
Mouse skin	TAC-1	✓	IRS	✗
HSPCs	Platelet	✓	LMPP	✗
Human brain	Cyc.	✓	ExDp	✗

Supplementary Table 5 | The comparison of velocity direction inferred by TIVelo for the original main path M based on origin node O, and the new main path M' based on new origin node O'. Root: If TIVelo can correctly select the expected root node R.

From Supplementary Table 5, TIVelo can correctly infer the expected root cluster in 8 out of 16 datasets when the new origin node O' is used. This highlights the importance of origin node selection

in TIVelo's performance, demonstrating that origin node selection can impact the accuracy of the final results.

To enhance the robustness of TIVelo, we integrated CytoTRACE2[18], a method for inferring cell potency from scRNA-seq data, into TIVelo to assist in origin node selection.

In TIVelo, after selecting the origin node O based on `scvelo.tl.terminal_states`, we can use CytoTRACE2 to calculate the median potency score P_i (from 0 to 1) for each cell cluster i. The results from CytoTRACE2 serve as an additional reference for refining origin node selection. The details are as follows.

1. If cell cluster O has the highest or lowest median potency scores across all clusters, O is selected as the origin node.
2. If cell cluster O does not have the highest or lowest median potency scores across all cell clusters, we select the cell cluster O'' with highest median potency score $P_{O''}$ as the origin node.

Supplementary Fig. 37 displays the box plots of potency scores of each cell cluster across the 16 datasets. We observed that in most datasets, the origin node O selected by `scvelo.tl.terminal_states` exhibits either the highest or lowest median potency score .

Furthermore, we denote the cluster with the second strongest signal in `scvelo.tl.terminal_states` as O', and the origin node after CytoTRACE2 refinement (for O') as O''. We then evaluated whether TIVelo could correctly infer the expected root node when O' and O'' were used as the origin node, respectively. From Supplementary Table 6, when O'' is selected as the origin node, TIVelo successfully infers the expected root node for all datasets except dentate gyrus 2 and mouse brain. In contrast, when O' is used as the origin node, only 8 out of the 16 datasets yield the correct root node inference.

Datasets	O'	Root	O''	Root
Pancreas	Beta	✓	Beta	✓
Dentate gyrus	Astrocytes	✗	nIPC	✓
Gastrulation	Erythoid 2	✓	Blood Progenitors 1	✓
Hindbrain (Oligo)	MFOLs	✓	MFOLs	✓
Intestinal organoid	Entrocytes	✓	Entrocytes	✓
Retina	PR	✓	Progenitor	✓
scNT	Time 60	✓	Time 60	✓
Dentate gyrus 2	Granule	✗	Granule	✗
Reprogramming	Cluster 3	✗	Cluster 9	✓
Hindbrain (GABA, Glial)	Differentiating GABA interneurons	✓	Neural stem cells	✓
RPE1-FUCCI	Leiden 1	✗	Leiden 0	✓
U2OS-FUCCI	Leiden 2	✓	Leiden 1	✓
Mouse brain	Deeper Layer	✗	Deeper Layer	✗
Mouse skin	IRS	✗	TAC-1	✓
HSPCs	LMPP	✗	Platelet	✓
Human brain	ExDp	✗	Cyc.	✓

Supplementary Table 6 | The comparison of TIVelo's results when O' or O'' is selected as the origin node. Root: if TIVelo can correctly infer the expected root node R when O' (O'') is selected as the origin node.

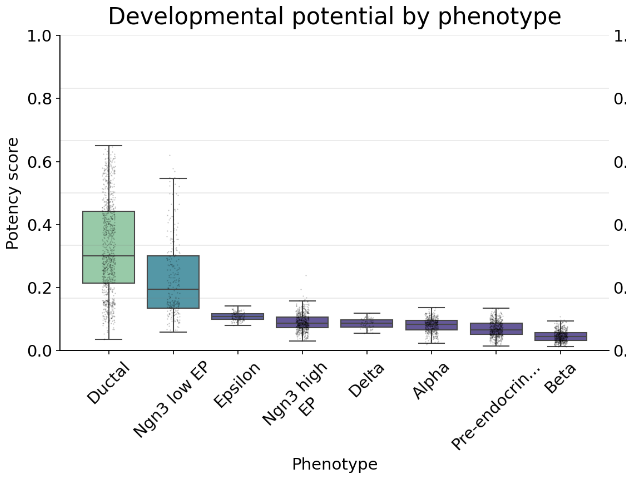

(a) Pancreas. O: Ductal.

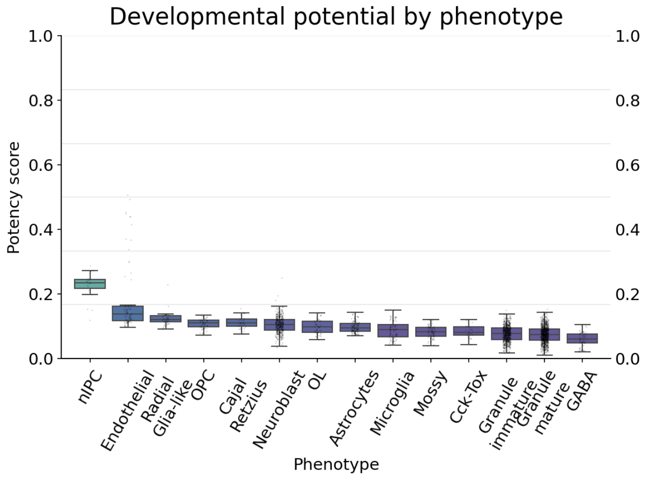

(b) Dentate gyrus. O: nIPC.

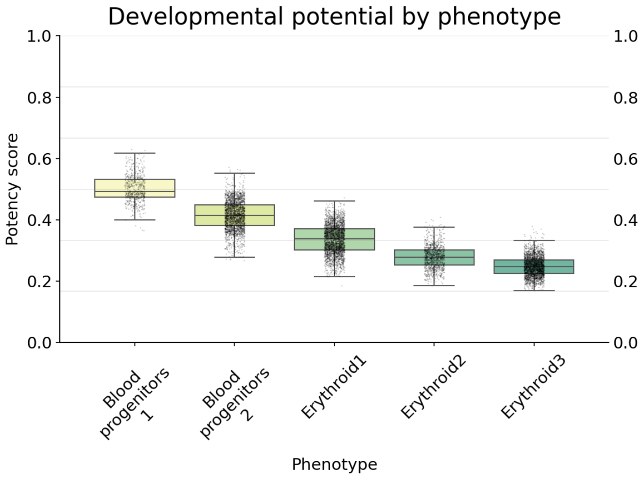

(c) Mouse gastrulation (erythroid). O: Blood progenitors 1.

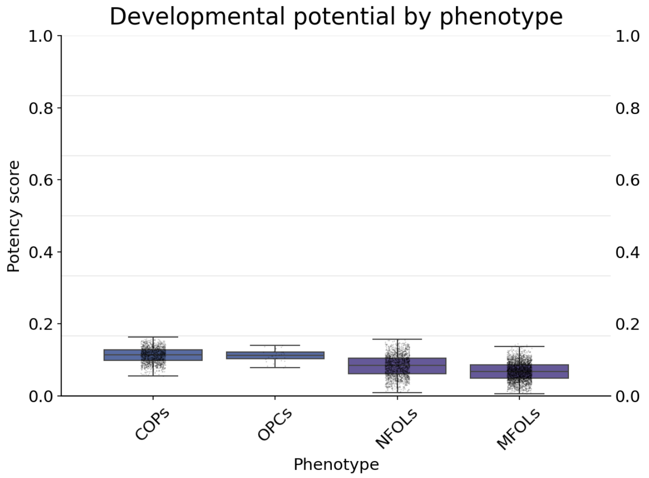

(d) Mouse hindbrain (Oligo). O: OPCs.

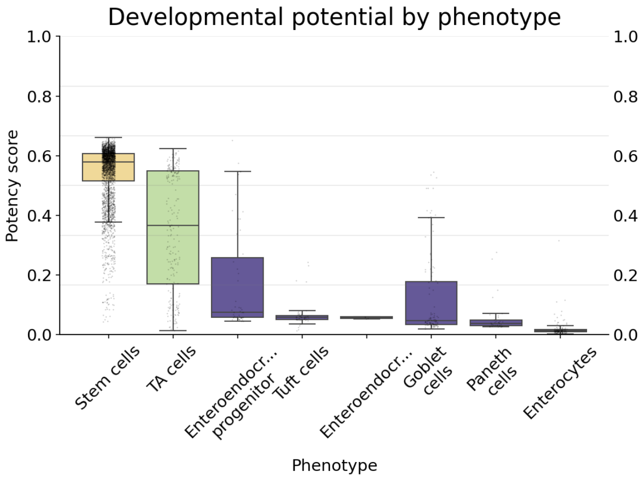

(e) Intestinal organoid. O: Paneth cells.

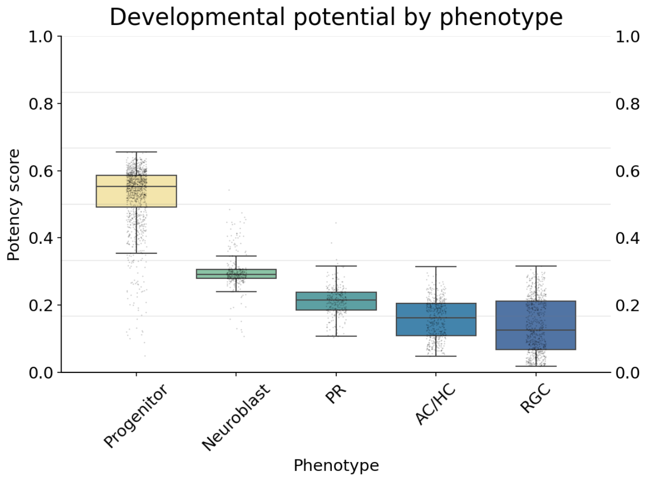

(f) Retina. O: AC/HC.

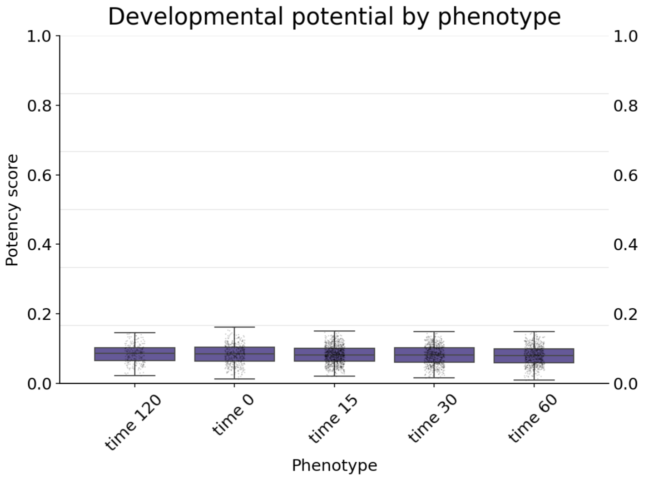

(g) scNT. O: Time 120.

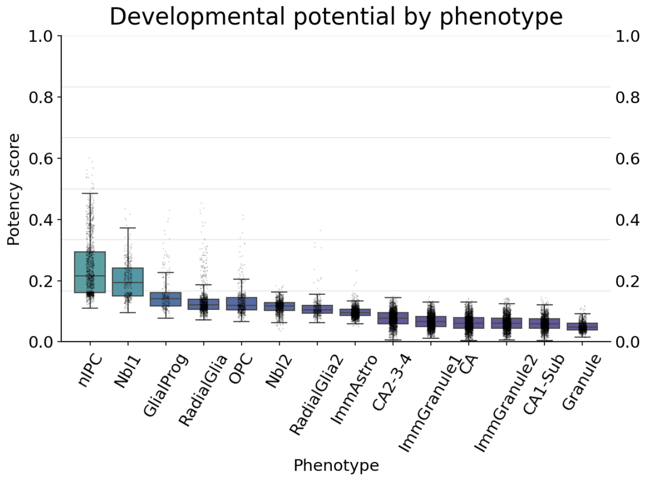

(h) Dentate gyrus 2. O: nIPC.

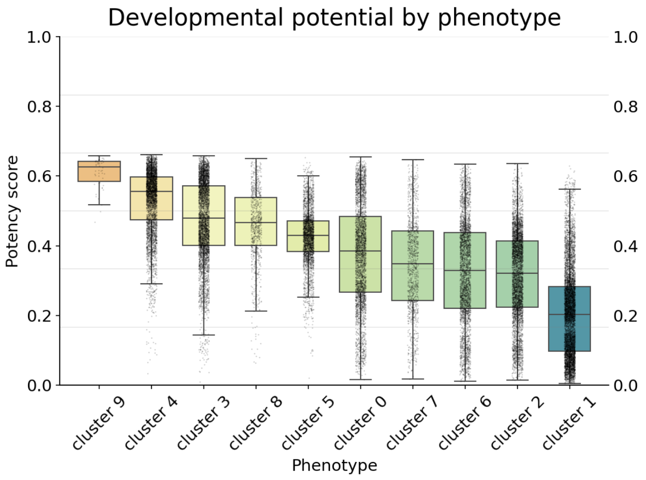

(i) Reprogramming. O: Cluster 9.

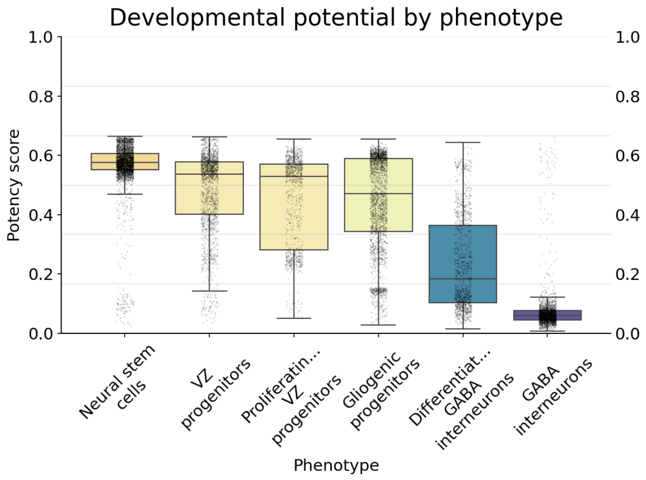

(j) Mouse hindbrain (GABA Glial). O: GABA interneurons.

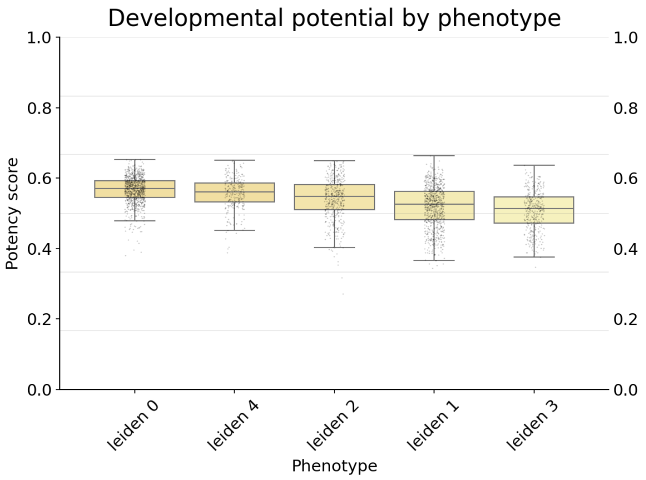

(k) RPE1-FUCCI. O: Leiden 3.

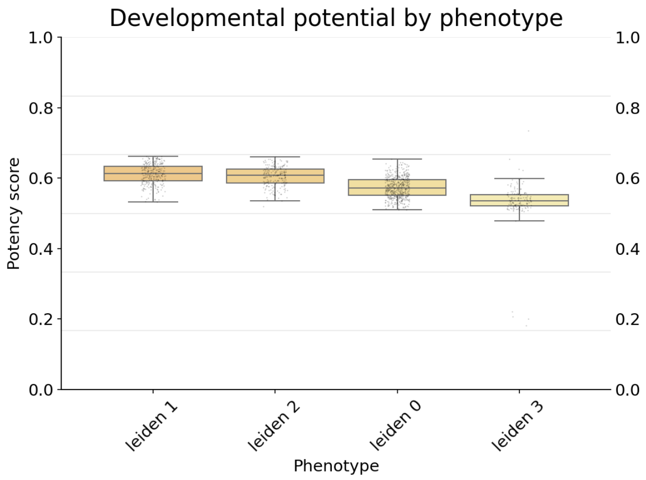

(l) U2OS-FUCCI. O: Leiden 1.

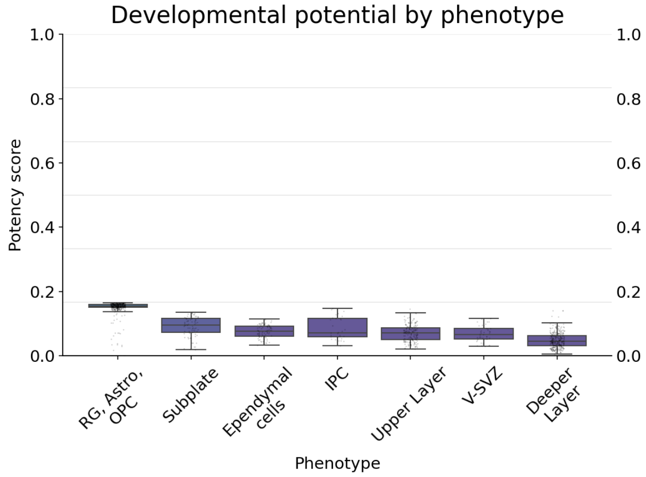

(m) Mouse brain. O: RG, Astro, OPC.

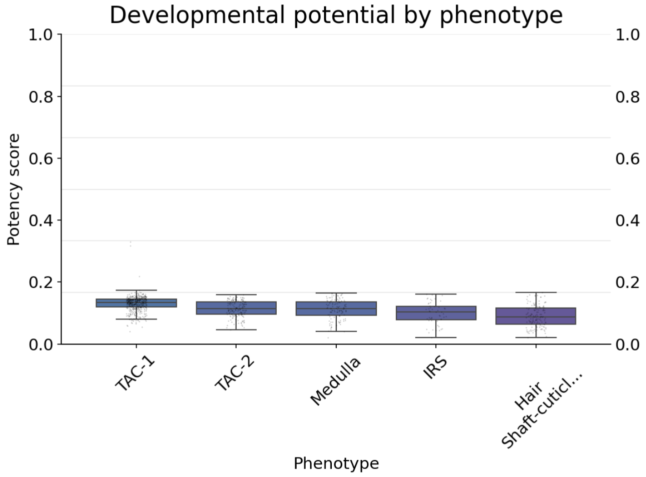

(n) Mouse skin. O: TAC-1.

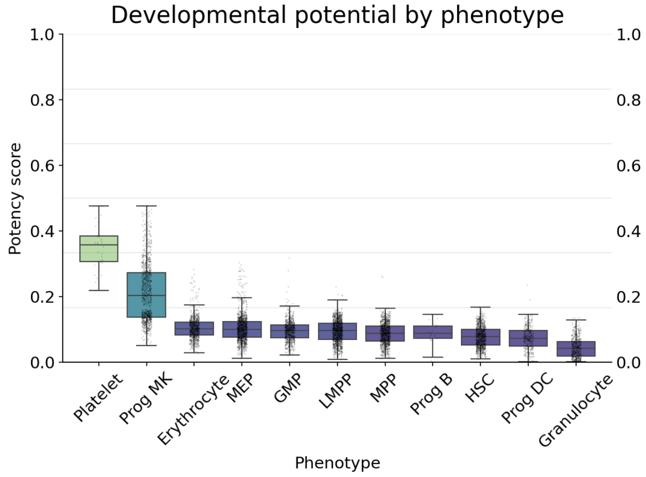

(o) HSPCs. O: Platelet.

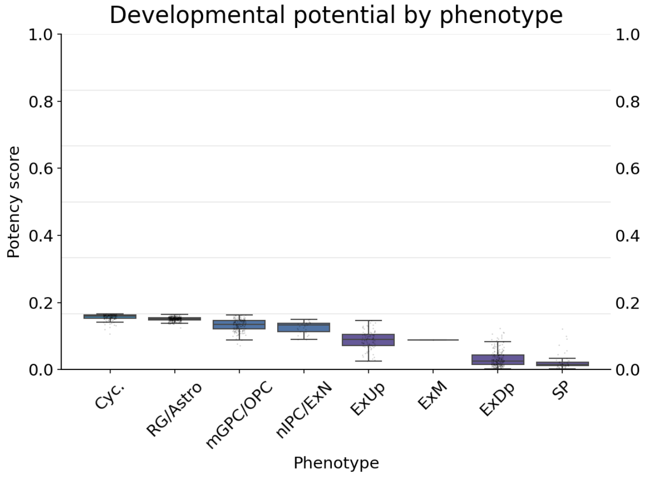

(p) Human brain. O: Cyc.

Supplementary Fig. 37 | Box plots of potency scores of each cell cluster across the 16 datasets, inferred by CytoTRACE2. In each box plot, the lower bound, centre and upper bound of the box plot stand for the first quartile (Q_1), median and the third quartile (Q_3) of the potency scores, respectively. The lower whisker and the upper whisker stand for $Q_1 - 1.5 \times \text{IQR}$ and $Q_3 + 1.5 \times \text{IQR}$, where $\text{IQR} = Q_3 - Q_1$.

Note 8 Root cell selection within root cluster

While TIVelo effectively infers velocity directions between clusters, the directionality within the root cluster can sometimes be ambiguous. The issue arises from the default way to select the root cell when applying the diffusion pseudotime: `adata.uns['iroot'] = np.flatnonzero(adata.obs['cell_types']=='Origin')[0]`. To address this issue, we have implemented a refined strategy for root cell selection. Specifically, we identify the cell within the inferred root cluster that has the longest distance (in the UMAP space) to the other clusters as the root cell. The resulting velocity streams show improved biological plausibility (Supplementary Fig. 38 and Supplementary Fig. 39), with this refinement particularly strengthening TIVelo's inference accuracy within the root cluster for mouse gastrulation (erythroid) and RPE1-FUCCI.

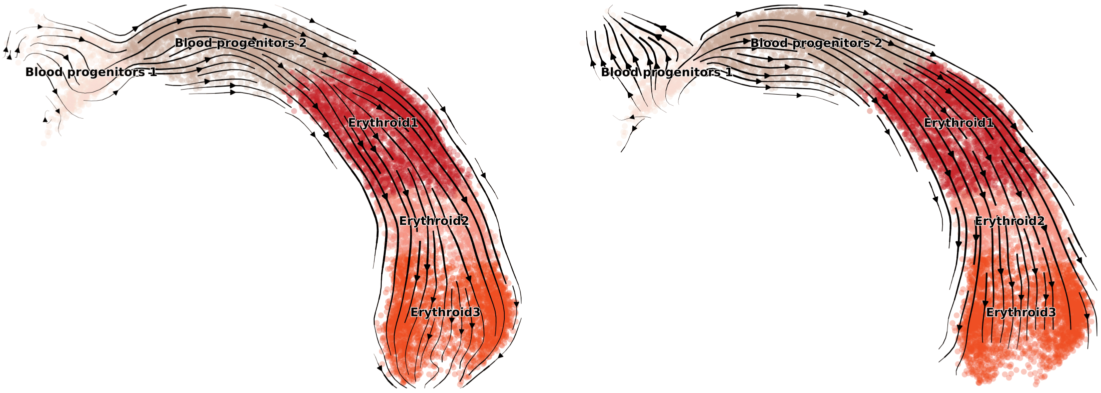

(a) Refined strategy for root cell selection (b) Root cell selection by default

Supplementary Fig. 38 | The comparison of velocity stream plots inferred by TIVelo for mouse gastrulation (erythroid), based on the refined strategy for root cell selection and root cell selection by default.

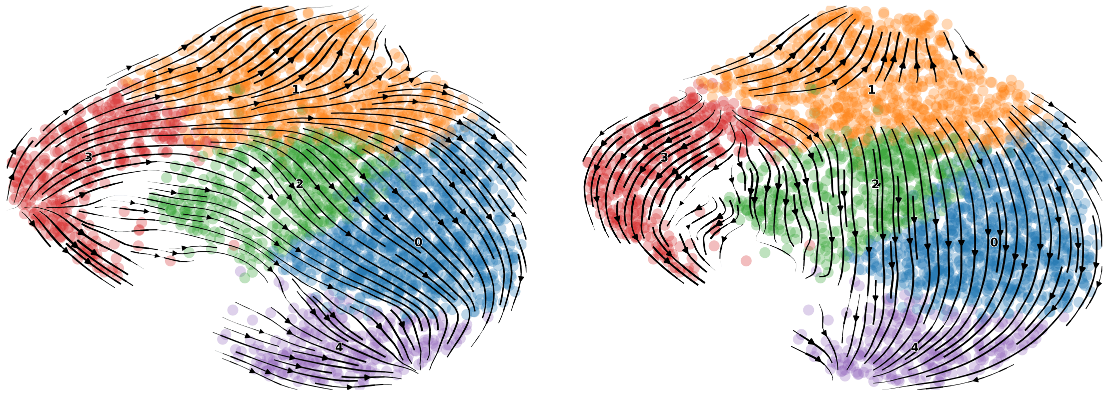

(a) Refined strategy for root cell selection (b) Root cell selection by default

Supplementary Fig. 39 | The comparison of velocity stream plots inferred by TIVelo for RPE1-FUCCI, based on the refined strategy for root cell selection and root cell selection by default. The numbers and colors represent different cell clusters.

Note 9 Randomness in scVelo's dynamical mode and veloVI

We produced the result of scVelo (dynamical mode) for dentate gyrus using the same sets of hyperparameters following the instructions in the scVelo tutorial (https://scvelo.readthedocs.io/en/stable/DynamicalModeling.html). The result of scVelo (dynamical mode) reproduced by our experiment is slightly different from the one given in the tutorial page (see Supplementary Fig. 40(a), red box). The observed differences may arise from inherent randomness in scVelo's dynamical mode. Notably, a similar velocity backward pattern is observed in the reproduction of scVelo (dynamical mode) for dentate gyrus from DeepVelo, as in Fig. 2(a) of the original DeepVelo paper[1].

Similar to scVelo, our experimental reproduction of veloVI yields results that are slightly different from those presented in Fig. 4(a) of the original veloVI paper (see Supplementary Fig. 40(b), red box). These discrepancies may also stem from the inherent randomness of the veloVI algorithm.

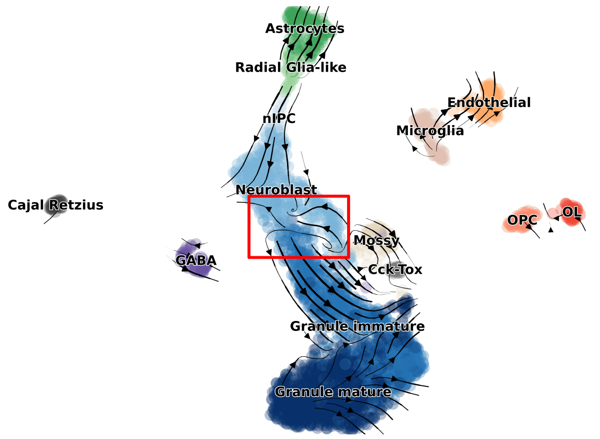

(a) scVelo (dynamical mode) from our experiment

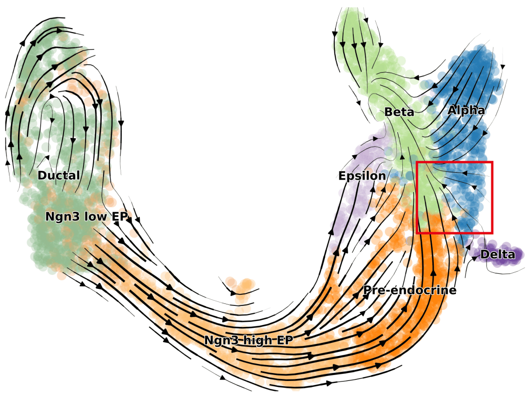

(b) veloVI from our experiment

Supplementary Fig. 40 | (a) Velocity stream plots inferred by scVelo (dynamical mode) for dentate gyrus in our experiment. (b) Velocity stream plots inferred by veloVI for pancreas in our experiment.

Note 10 Mode selection of UniTVelo

In UniTVelo[19], the preferred mode for datasets with cell cycle phase included (pancreas, retina, RPE1-FUCCI and U2OS-FUCCI) or with sparse cell types included (dentate gyrus) should be the independent mode (mode 2). The comparison of velocity stream plots produced by both UniTVelo mode 1 and mode 2 for such datasets is presented in Supplementary Fig. 41-45. In our quantitative comparison, we used UniTVelo mode 2 for these five datasets, as shown in Fig. 5 and Fig. 7.

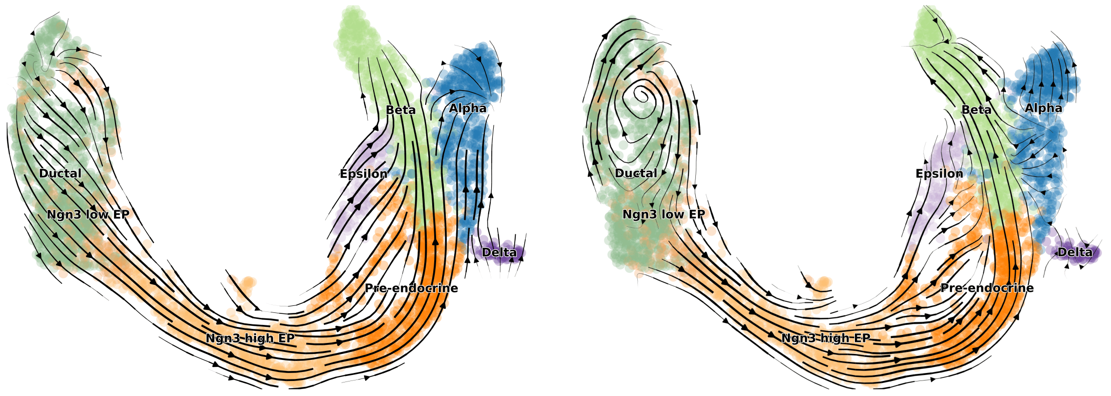

(a) UniTVelo mode 1 (b) UniTVelo mode 2

Supplementary Fig. 41 | Comparison of velocity stream plots inferred by UniTVelo mode 1 and mode 2 for pancreas.

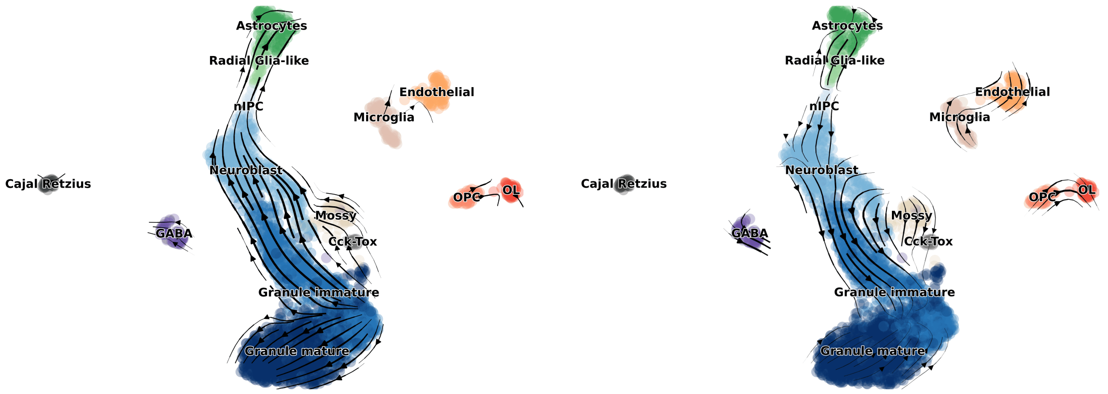

(a) UniTVelo mode 1 (b) UniTVelo mode 2

Supplementary Fig. 42 | Comparison of velocity stream plots inferred by UniTVelo mode 1 and mode 2 for dentate gyrus.

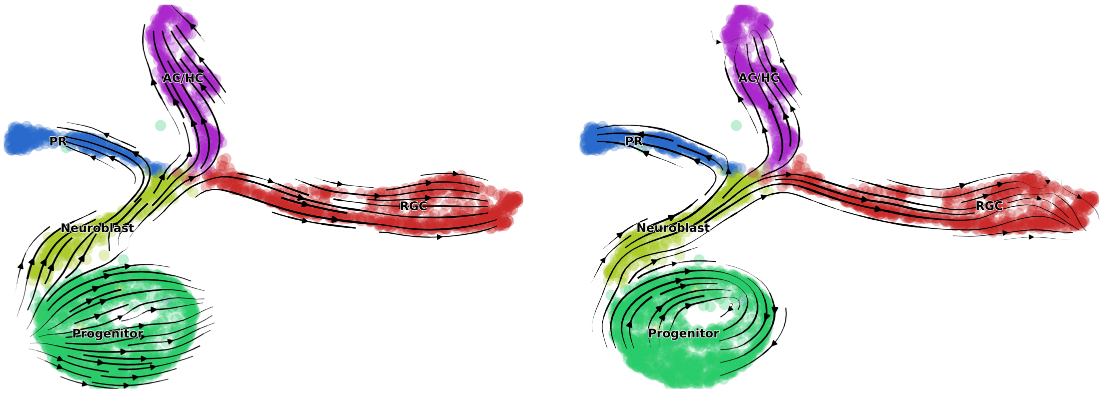

(a) UniTVelo mode 1 (b) UniTVelo mode 2

Supplementary Fig. 43 | Comparison of velocity stream plots inferred by UniTVelo mode 1 and mode 2 for retina.

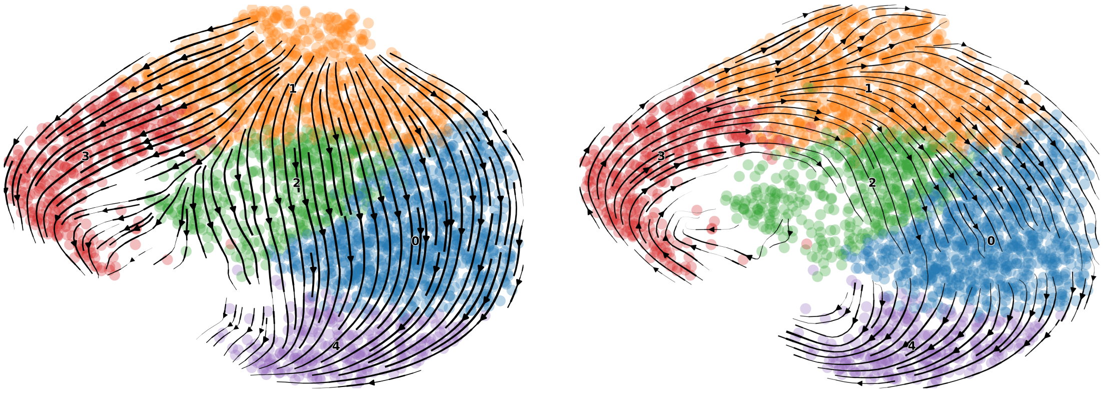

(a) UniTVelo mode 1 (b) UniTVelo mode 2

Supplementary Fig. 44 | Comparison of velocity stream plots inferred by UniTVelo mode 1 and mode 2 for RPE1-FUCCI.

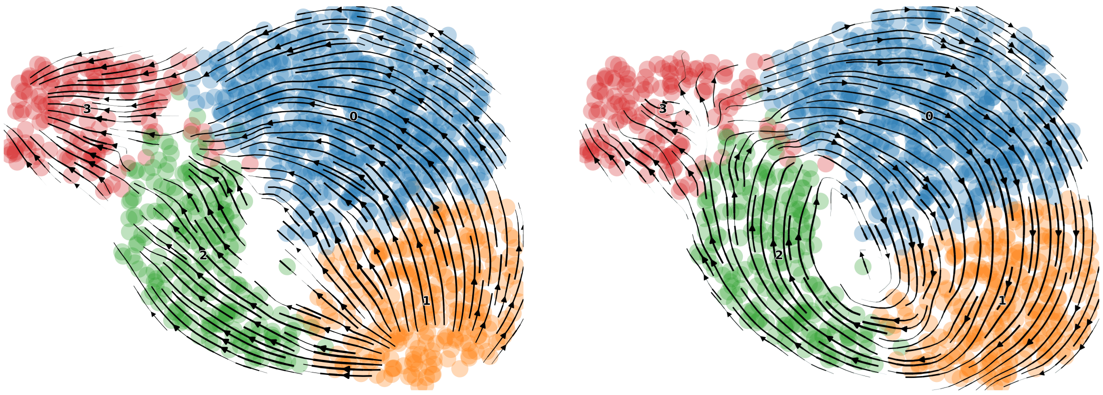

(a) UniTVelo mode 1 (b) UniTVelo mode 2

Supplementary Fig. 45 | Comparison of velocity stream plots inferred by UniTVelo mode 1 and mode 2 for U2OS-FUCCI.

Note 11 Root cluster selection of UniTVelo

For the intestinal organoid dataset (Fig. 4 of the original UniTVelo paper[19]), UniTVelo originally set `velo_config.IROOT` to the expected root cell cluster (Stem cells) (https://github.com/StatBiomed/UniTVelo/blob/main/notebooks/Figure4_IntestinalOrganoid.ipynb), which leads to unfairness when comparing with other methods that do not use this information (Supplementary Fig. 46(a)). To ensure fairness, we set `velo_config.IROOT` to `None` by default (Supplementary Fig. 46(b)).

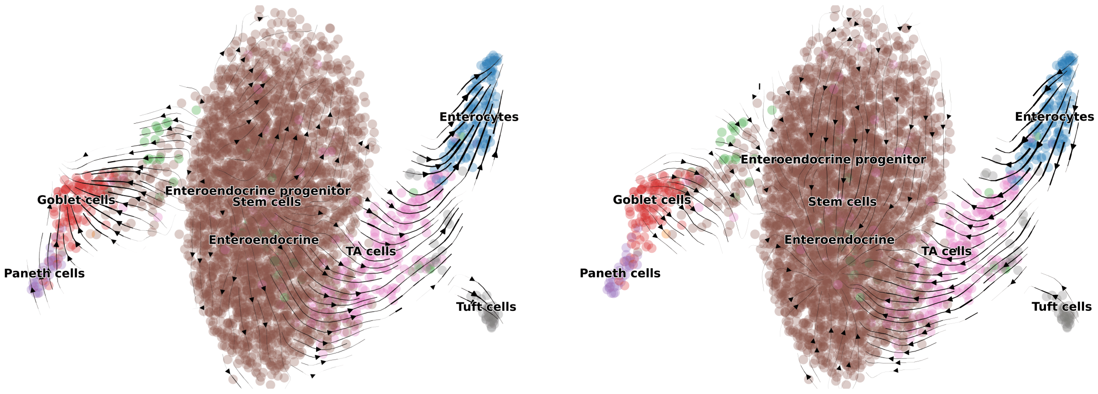

(a) UniTVelo with root cell cluster (b) UniTVelo without root cell cluster

Supplementary Fig. 46 | Comparison of velocity stream plots inferred by UniTVelo with and without root cell cluster given for intestinal organoid.

Note 12 Reproduction of cellDancer results

There are some discrepancies between the results from our reproduction of cellDancer (pancreas, mouse gastrulation (erythroid) and dentate gyrus 2) and in original cellDancer paper[2] (Fig. 4, Fig. 2, Fig. 3). These discrepancies stem from two key factors:

Firstly, for consistency across methods, we used the `scvelo.pl.velocity_embedding_stream` function in the scVelo package to visualize the results from all methods, including cellDancer. We note that the built-in visualization in cellDancer (`celldancer.cdplt.scatter_gene`) uses Bezier curves to generate smoother and more aesthetically pleasing arrows, which may contribute to the observed differences.

To provide additional context, we include both visualization approaches from scVelo and cellDancer. Comparisons of the two visualization methods for pancreas, mouse gastrulation (erythroid) and dentate gyrus 2 are shown in Supplementary Fig. 47, Supplementary Fig. 48, and Supplementary Fig. 49, respectively.

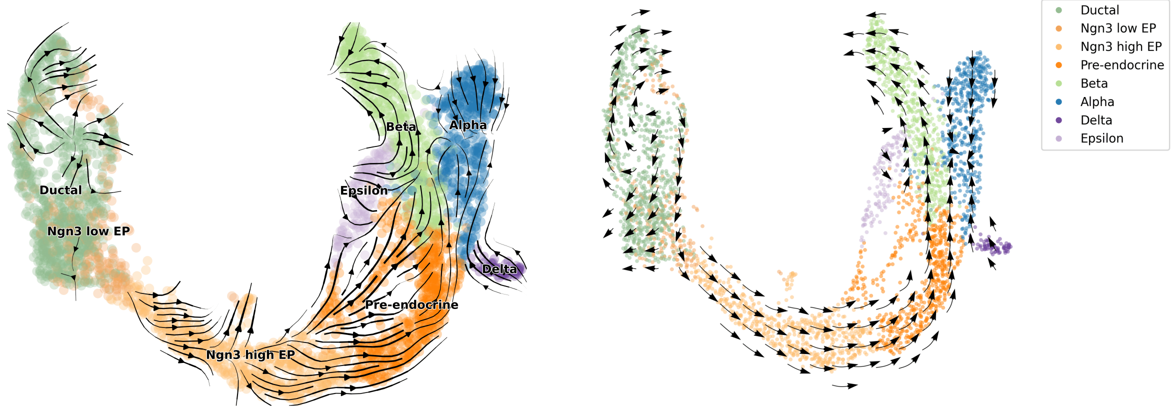

(a) Velocity stream inferred by cellDancer, using visualization from scVelo (b) Velocity stream inferred by cellDancer, using cellDancer built-in visualization

Supplementary Fig. 47 | Comparison of velocity stream plots inferred by cellDancer for pancreas, through scVelo visualization and cellDancer built-in visualization, respectively.

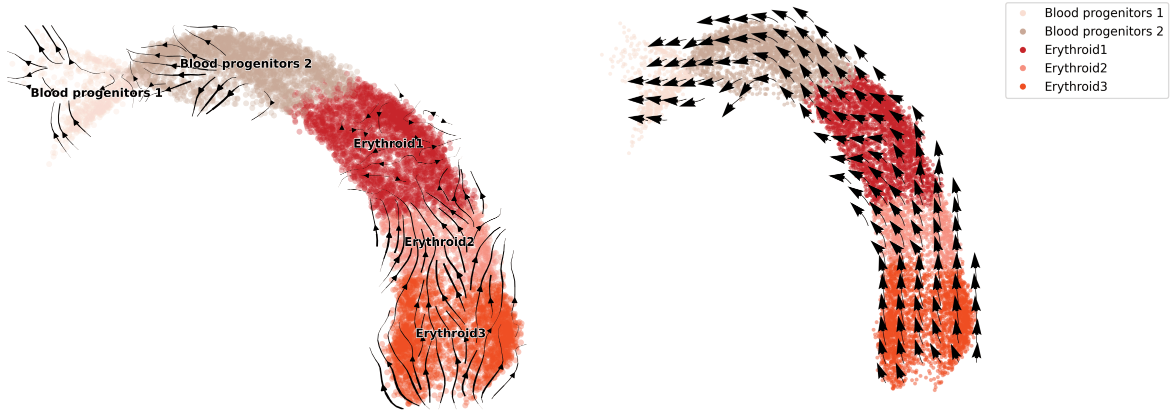

(a) Velocity stream inferred by cellDancer, using visualization from scVelo (b) Velocity stream inferred by cellDancer, using cellDancer built-in visualization

Supplementary Fig. 48 | Comparison of velocity stream plots inferred by cellDancer for mouse gastrulation (erythroid), through scVelo visualization and cellDancer built-in visualization, respectively.

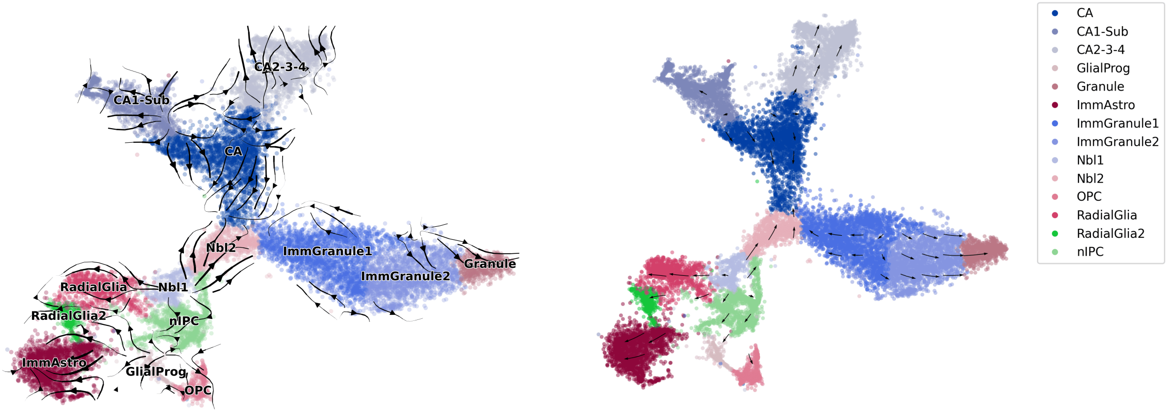

(a) Velocity stream inferred by cellDancer, using visualization from scVelo

(b) Velocity stream inferred by cellDancer, using cellDancer built-in visualization

Supplementary Fig. 49 | Comparison of velocity stream plots inferred by cellDancer for dentate gyrus 2, through scVelo visualization and cellDancer built-in visualization, respectively.

Secondly, cellDancer did not use the same preprocessing procedure and hyperparameters in the model for different datasets in the tutorial (`https://guangyuwanglab2021.github.io/cellDancer_website/index.html`). Specifically,

1. **Pancreas**
   - Using `permutation_ratio`=0.5 in `celldancer.velocity` function, which is the sampling ratio of cells in each epoch when training each gene.
   - Using 200 neighboring cells for calculating the transition probability matrix for each cell (which means `projection_neighbor_size`=200 in `celldancer.compute_cell_velocity` function).
2. **Mouse gastrulation (erythroid)**
   - Selecting cells from cell type haemato-endothelial progenitors, blood progenitors 1/2, and erythroid 1/2/3 in a large mouse gastrulation dataset[20], which is different from the more commonly used smaller dataset (used by UniTVelo, DeepVelo, veloAE[21], etc.), with cells selected from blood progenitors 1/2 and erythroid 1/2/3.
   - Using 100 neighboring cells for first-moment calculation in preprocessing, (which means `n_neighbors`=100 in `scvelo.pp.moments` function, instead of 30 by default).
   - Using `permutation_ratio`=0.125 in `celldancer.velocity` function.
   - Using 10 neighboring cells for calculating the transition probability matrix for each cell.
3. **Dentate gyrus 2**
   - Without using `scvelo.pp.filter_and_normalize` to filter genes.
   - Using `permutation_ratio`=0.1 in `celldancer.velocity` function.
   - Using 100 neighboring cells for calculating the transition probability matrix for each cell.

For a fair comparison, we adopted the same preprocessing procedure and hyperparameters in the model for all datasets when we implemented cellDancer:

1. Using scvelo.pp.filter_and_normalize and scvelo.pp.moments for preprocessing with default arguments. Using "velocity genes" defined in UniTVelo for RNA velocity analysis.
2. Inferring RNA velocity for each cell following the instruction in the tutorial for mouse gastrulation (erythroid) (https://guangyuwanglab2021.github.io/cellDancer_website/notebooks/case_study_gastrulation.html):
   - Using celldancer.velocity with permutation_ratio=0.125.
   - Using celldancer.compute_cell_velocity with projection_neighbor_size=10.

For the three datasets pancreas, mouse gastrulation (erythroid) and dentate gyrus 2, we also reproduced the results by cellDancer, following the preprocessing procedure and the hyperparameter settings in cellDancer's tutorial. We can reproduce the same result as in the cellDancer paper, as shown in Supplementary Fig. 50.

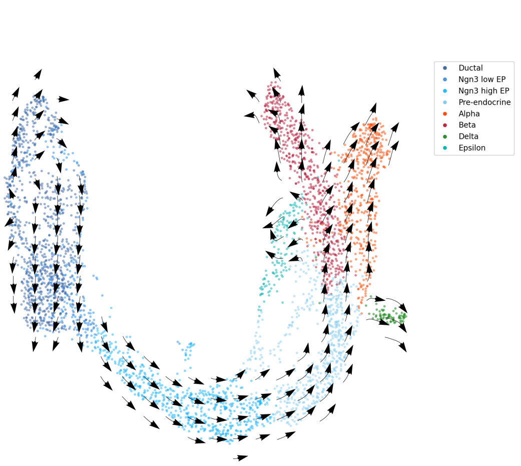

(a) Velocity stream inferred by cellDancer for pancreas, following the tutorial of cellDancer

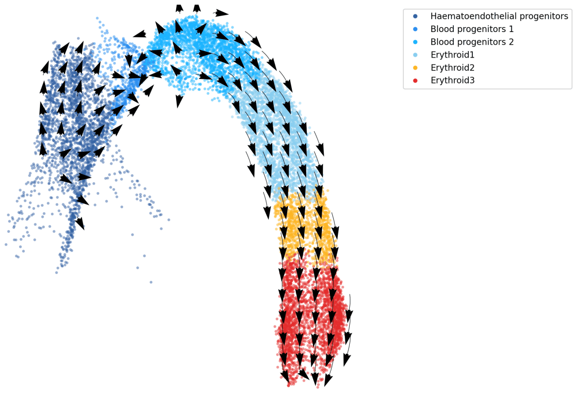

(b) Velocity stream inferred by cellDancer for mouse gastrulation (erythroid), following the tutorial of cellDancer

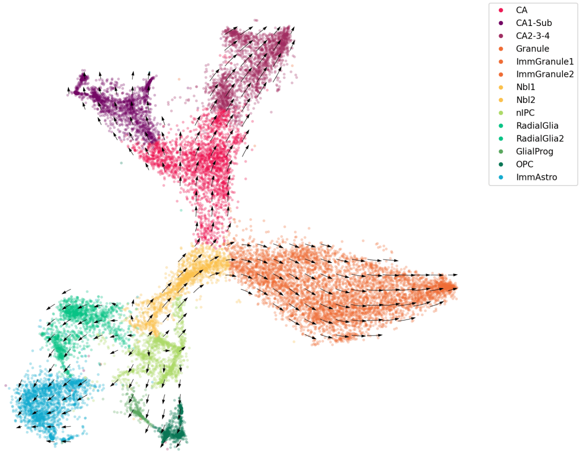

(c) Velocity stream inferred by cellDancer for dentate gyrus 2, following the tutorial of cellDancer

Supplementary Fig. 50 | Velocity stream plots inferred by cellDancer for pancreas, mouse gastrulation (erythroid) and dentate gyrus 2, following the preprocessing procedure and the hyperparameter settings in the tutorial of cellDancer.

We compared the performance of cellDancer (in our experiments with uniform settings), cellDancer (following the different settings for different datasets in cellDancer's tutorial) and TIVelo for these three datasets using three quantitative metrics we used in the manuscript, as shown in Supplementary Table 7. While some specific dataset and metric combinations show improvement when following the settings in the tutorial of cellDancer, others actually perform worse. In contrast, TIVelo consistently outperforms cellDancer across all datasets and metrics.

Metrics	Methods	Pancreas	Gastrulation	Dentate gyrus 2
CBDir (Gene space)	cellDancer (uniform settings)	0.0583	-0.2989	0.0404
	cellDancer (tutorial settings)	0.0720	0.0271	0.0035
	TIVelo	0.4850	0.6164	0.4265
TransCosine	cellDancer (uniform settings)	0.0479	0.0001	0.0001
	cellDancer (tutorial settings)	0.2169	0.1258	0.0547
	TIVelo	0.4745	0.4436	0.3742
VeloCoh	cellDancer (uniform settings)	-0.2845	-0.1609	-0.1316
	cellDancer (tutorial settings)	-0.4270	-0.2383	0.0005
	TIVelo	0.1696	0.2504	0.1552

Supplementary Table 7 | The comparison of cellDancer (in our experiments with uniform settings), cellDancer (following the settings in cellDancer's tutorial) and TIVelo through three quantitative metrics for pancreas, mouse gastrulation (erythroid) and dentate gyrus 2.

References

1. Cui, H. et al. DeepVelo: deep learning extends RNA velocity to multi-lineage systems with cell-specific kinetics. *Genome Biol.* **25**, 27 (2024).

2. Li, S. et al. A relay velocity model infers cell-dependent RNA velocity. *Nat. Biotechnol.* **42**, 99-108 (2024).

3. Weiler, P., Lange, M., Klein, M., Pe'er, D., & Theis, F. CellRank 2: unified fate mapping in multiview single-cell data. *Nat. Methods* **21**, 1196-1205 (2024).

4. Velten, L. et al. Human haematopoietic stem cell lineage commitment is a continuous process. *Nat. Cell Biol.* **19**, 271-281 (2017).

5. Park, S. et al. The protein disulfide isomerase AGR2 is essential for production of intestinal mucus *Proc. Natl Acad. Sci.* **106**, 6950-6955 (2009).

6. Zhao, F. et al. Disruption of Paneth and goblet cell homeostasis and increased endoplasmic reticulum stress in Agr2-/- mice. *Dev. Biol.* **338**, 270-279 (2010).

7. Bennett, E. P. et al. Control of mucin-type O-glycosylation: a classification of the polypeptide GalNAc-transferase gene family. *Glycobiology* **22**, 736-756 (2012).

8. Fox, R. M., Hanlon, C. D.,& Andrew, D. J. The CrebA/Creb3-like transcription factors are major and direct regulators of secretory capacity. *J. Cell Biol.* **191**, 479-492 (2010).

9. Sheng, Y. H. et al. The MUC13 cell-surface mucin protects against intestinal inflammation by inhibiting epithelial cell apoptosis. *Gut* **60**, 1661-1670 (2011).

10. Gao, F. et al. Hspa5 Deficiency Blocks Intestinal Enterocyte Differentiation in Graft-Versus-Host Disease. *Blood* **142**, 2049 (2023).

11. Kaemmerer, E. et al. Increased levels of deleted in malignant brain tumours 1 (DMBT1) in active bacteria-related appendicitis. *Histopathology* **60**, 561-569 (2012).

12. McConnell, R. E., & Tyska, M. J. Myosin-1a powers the sliding of apical membrane along microvillar actin bundles. *J. Cell Biol.* **177**, 671-681 (2007).

13. Cao, Z. Q., & Guo, X. L. The role of galectin-4 in physiology and diseases. *Protein Cell* **7**, 314-324 (2016).

14. Lange, M. et al. CellRank for directed single-cell fate mapping. *Nat. Methods* **19**, 159-170 (2022).

15. Silva, J. et al. Ribosome impairment regulates intestinal stem cell identity via ZAKα activation. *Nat. Commun.* **13**, 4492 (2022).

16. Gayoso, A. et al. Deep generative modeling of transcriptional dynamics for RNA velocity analysis in single cells. *Nat. Methods* **21**, 50-59 (2024).

17. Bergen, V., Lange, M., Peidli, S., Wolf, F. A., & Theis, F. J. Generalizing RNA velocity to transient cell states through dynamical modeling. *Nat. Biotechnol.* **38**, 1408-1414 (2020).

18. Kang, M. et al. Mapping single-cell developmental potential in health and disease with interpretable deep learning. Preprint at https://www.biorxiv.org/content/10.1101/2024.03.19.585637v1 (2024).

19. Gao, M., Qiao, C., & Huang, Y. UniTVelo: temporally unified RNA velocity reinforces single-cell trajectory inference. *Nat. Commun.* **13**, 6586 (2022).

20. Pijuan-Sala, B. et al. A single-cell molecular map of mouse gastrulation and early organogenesis. *Nature* **566**, 490-495 (2019).

21. Qiao, C., & Huang, Y. Representation learning of RNA velocity reveals robust cell transitions. *Proc. Natl Acad. Sci.* **118**, e2105859118 (2021).
